# From environmental awareness to ecological behavior: a bibliometric mapping of the literature

**DOI:** 10.3389/frma.2026.1906919

**Published:** 2026-09-18

**Authors:** Fikret Kayhalak, Suat Karaküçük

**Affiliations:** 1 Department of Recreation, Faculty of Sports Sciences, Istanbul Gelisim University, Istanbul, Türkiye; 2 Department of Sports Management, Faculty of Sports Sciences, Yozgat Bozok University, Yozgat, Türkiye

**Keywords:** bibliometric laws, bibliometrics, environmental awareness, environmental concern, environmental knowledge, pro-environmental behavior, science mapping, thematic evolution

## Abstract

This study maps the intellectual, conceptual, and social structure of the research literature connecting environmental awareness to ecological behavior through a systematic bibliometric analysis. Bibliographic records were retrieved from Web of Science (WoS) Core Collection and Scopus using a comprehensive dual-database search strategy, yielding 934 unique publications after duplicate removal. A database complementarity analysis revealed that 222 records (23.8%) were exclusive to Scopus, with Scopus-only records exhibiting a lower mean citation count (20.3 vs. 40.5) and earlier mean publication year (2017 vs. 2019) compared to WoS records, confirming the necessity of dual-database indexing. All analyses were conducted in R using the bibliometrix package. Findings document a substantial acceleration in annual production, particularly after 2016, with a peak of 132 publications in 2025. Bradford's Law analysis identified 39 core journals (33.2% of publications), with a zone multiplier of 1:4.4:7.9—deviating from the classical geometrical prediction—consistent with a disciplinarily heterogeneous field. Lotka's Law analysis revealed that 88.94% of authors contributed only one publication, significantly exceeding the theoretical expectation of 65.47% (residual = +0.2347), signaling an active but structurally fragmented contributory base. Keyword sensitivity analysis confirmed that the thematic structure—anchored on pro-environmental behavior, attitudes, environmental attitudes, environmental concern, and sustainability—remained stable across minimum co-occurrence thresholds of ≥3, ≥5, and ≥7, demonstrating high robustness. Thematic evolution analysis revealed three distinct developmental phases: an early foundation stage (pre-2010) dominated by knowledge and attitude frameworks, a consolidation stage (2011–2020) centered on behavioral mechanisms, and an acceleration stage (2021–2026) integrating sustainability, digital, and cross-cultural dimensions. Together, these findings suggest the field may be growing and theoretically shifting from antecedent-focused to mechanism- and application-oriented inquiry, with citation and keyword patterns appearing consistent with structural consolidation.

## 1 Introduction

The deepening of global environmental challenges has placed the behavioral dimensions of sustainability at the forefront of scientific inquiry. Climate change, biodiversity loss, natural resource depletion, and ecological degradation are no longer treated solely as technical or policy problems; increasingly, they are understood as problems of individual and collective behavior, requiring sustained attention to the psychological, social, and educational processes through which people form environmental dispositions and translate them into action (Stern, 2000; Kollmuss and Agyeman, 2002; Steg and Vlek, 2009). Within this agenda, the construct of environmental awareness—broadly encompassing environmental knowledge, environmental concern, environmental attitudes, and ecological sensitivity—has emerged as a foundational but conceptually contested antecedent of pro-environmental behavior (Gifford and Nilsson, 2014; Geiger et al., 2019).

Pro-environmental behavior (PEB) is defined as a constellation of actions that minimize harm to, or actively benefit, the natural environment (Kaiser et al., 1999; Stern, 2000; Lange and Dewitte, 2019). It is a multidimensional construct encompassing energy conservation, recycling, sustainable consumption, transport choices, nature preservation, civic engagement, and green purchasing, among others (Steg and Vlek, 2009; Turaga et al., 2010). The relationship between environmental awareness and PEB has been studied across environmental psychology, education, consumer behavior, tourism, public health, and sustainability science—generating a body of literature that is simultaneously rich in empirical depth and fragmented in theoretical coherence (Bamberg and Möser, 2007; Gifford, 2011; Lacroix et al., 2019). Crucially, this body of evidence marks a paradigmatic shift: the field has progressively moved away from awareness-centered explanatory models toward mechanism- and intervention-oriented research programmes, a transition that is visible in both the conceptual vocabulary and the authorial structure of the literature (Cobo et al., 2011; Mukherjee et al., 2022).

Despite substantial empirical accumulation, several important structural questions about this literature remain unanswered. Which journals constitute the disciplinary core of the field? How is authorship concentrated, and does it follow classical bibliometric laws? What are the dominant and emerging conceptual themes, and have they shifted over time? To what extent does the structure of the field depend on the database from which records are drawn? These questions are not addressable through narrative or systematic review alone; they require the analytical tools of bibliometrics and science mapping (Donthu et al., 2021; Aria and Cuccurullo, 2017).

Bibliometric analysis has gained substantial traction as a method for mapping the structural, intellectual, and social dimensions of research fields (Zupic and Cater, 2015; Mukherjee et al., 2022). Unlike narrative reviews, it provides reproducible, quantitative characterisations of knowledge production: citation patterns, authorial networks, source concentration, keyword co-occurrence structures, and thematic trajectories (van Eck and Waltman, 2010; Cobo et al., 2011). In rapidly growing, interdisciplinary fields prone to conceptual fragmentation—precisely the characteristics of the environmental awareness–ecological behavior nexus—bibliometric mapping offers irreplaceable structural visibility.

Several prior bibliometric studies have examined domains adjacent to or partially overlapping with the present research. Peng and Bai (2021) conducted a bibliometric analysis of environmental psychology broadly construed, documenting growth patterns and thematic clusters from 1991 to 2020. More recently, Zhu et al. (2021) applied CiteSpace to 818 Web-of-Science records on environmental behavior (2002–2020), identifying dominant research hotspots and disciplinary clusters. Goyal and Goyal (2024) employed SciMAT and VOSviewer to trace the evolution of pro-environmental behavior research across three decades using 1,824 Scopus records (1993–2022), identifying ecotourism, sustainable consumption, and CSR as dominant emerging themes. While these contributions are valuable, they share three common limitations that the present study is designed to address: (1) each relies on a single database (either WoS or Scopus), precluding systematic assessment of database complementarity; (2) none validates the structural regularities of the field against classical bibliometric laws (Bradford's Law, Lotka's Law); and (3) none specifically maps the conceptual intersection of environmental awareness constructs (environmental knowledge, concern, attitudes, consciousness) and ecological/pro-environmental behavior as a unified construct nexus. The present study addresses all three gaps by combining dual-database coverage, bibliometric law validation, and keyword sensitivity testing within a single integrated analysis. Beyond methodological integration, this study generates substantive knowledge about the field itself: by revealing that single-database retrieval systematically excludes a structurally distinct segment of the literature (23.8% Scopus-exclusive records with earlier publication years and lower citation counts), it demonstrates that the intellectual map of this domain has been empirically incomplete in prior reviews. Furthermore, the validation of bibliometric law deviations—particularly the excess of transient authors relative to Lotka's theoretical prediction—provides the first quantitative characterization of the field's contributory fragmentation, a structural feature with direct implications for how future reviews, funding priorities, and research training programmes in environmental behavior should be designed.

Accordingly, this study pursues four objectives: (1) to characterize the temporal, source, and authorial structure of the environmental awareness–ecological behavior literature; (2) to map its conceptual and thematic architecture through keyword co-occurrence and thematic evolution analysis; (3) to validate the structural regularities of the field using Bradford's and Lotka's laws; and (4) to assess the methodological robustness of the findings through database complementarity and keyword sensitivity analyses. These objectives are operationalised through four research questions. RQ1: How has annual scientific production in the environmental awareness–ecological behavior literature evolved over time, and which sources, authors, and countries have shaped its structural development? RQ2: Does the structural organization of the field conform to classical bibliometric laws (Bradford's Law, Lotka's Law), and what do deviations from these laws indicate about the field's maturation stage? RQ3: What is the thematic architecture of the field, how has it evolved across distinct developmental phases, and which themes function as motors, anchors, or emerging drivers? RQ4: To what extent are the structural and thematic findings robust to key methodological choices—specifically, database selection and keyword co-occurrence threshold?

## 2 Methods

### 2.1 Research design

This study employs a quantitative bibliometric research design combining performance analysis and science mapping (Aria and Cuccurullo, 2017; Donthu et al., 2021). Performance analysis quantifies the productivity and impact of scholarly units (journals, authors, countries); science mapping visualizes the intellectual and conceptual structure of a field through network and cluster analysis (Cobo et al., 2011; van Eck and Waltman, 2010). The integration of these two approaches allows both a macroscopic view of knowledge production and a fine-grained characterization of conceptual architecture (Zupic and Cater, 2015; Mukherjee et al., 2022). Because the environmental awareness–ecological behavior literature spans environmental psychology, education, consumer behavior, and sustainability science, bibliometric mapping provides an appropriate and efficient framework for identifying structural regularities and thematic trajectories that would otherwise be obscured by disciplinary boundaries.

### 2.2 Data sources

Data were retrieved from two complementary bibliographic databases: Web of Science (WoS) Core Collection and Scopus. The choice to use both databases reflects the known differences in their indexing scope, disciplinary coverage, and temporal depth. WoS applies more selective quality criteria and is particularly strong in natural sciences and psychology; Scopus provides broader coverage across social sciences, education, and business (Mongeon and Paul-Hus, 2016). Prior research has demonstrated that exclusive reliance on either database risks systematic underrepresentation of subdisciplines—a particularly acute concern in interdisciplinary fields such as the present one. The complementarity of the two databases was formally evaluated as part of this study (see Section 2.4.1).

### 2.3 Search strategy and data collection

The search strategy was designed to capture the full terminological diversity of the environmental awareness–ecological behavior nexus. Two conceptual clusters were operationalised: (1) environmental awareness, represented by “environmental awareness,” “ecological awareness,” “environmental consciousness,” “environmental concern,” “environmental knowledge,” and “environmental attitude^*^”; and (2) ecological/pro-environmental behavior, represented by “ecological behavio?r,” “pro-environmental behavio?r,” “environmental behavio?r,” “sustainable behavio?r,” and “green behavio?r.” The wildcard “behavio?r” captured both American and British English spellings. Searches were applied to title, abstract, and keyword fields. Only articles and review papers were included, consistent with established bibliometric practice (Donthu et al., 2021). Book chapters, conference proceedings, and gray literature were excluded because their indexing in WoS and Scopus is incomplete and inconsistent, which would introduce systematic retrieval bias into the performance and network analyses. Early access articles indexed at the date of extraction (6 April 2026) were retained, as they represent formally accepted and citable contributions; their inclusion is consistent with the decision to treat the 2025 production count as fully indexed. No language restriction was applied at the search stage; however, both WoS and Scopus index predominantly English-language publications, which means that non-English contributions are likely underrepresented in the dataset. Data were extracted on 6 April 2026, yielding 712 records from WoS and 762 records from Scopus.

### 2.4 Data cleaning and preparation

A multi-stage data cleaning protocol was applied. In the first stage, WoS and Scopus records were merged into a single bibliographic dataset. In the second stage, duplicate records were identified and removed using DOI- and title-based matching, eliminating 540 duplicates and retaining 934 unique publications; ambiguous cases where DOIs were absent or titles differed marginally were resolved through manual inspection by the research team. In the third stage, incomplete or inconsistent bibliographic fields were reviewed. In the fourth stage, keyword standardization was performed: synonymous terms were merged, spelling variants were unified, and semantic fragmentation was minimized (e.g., “pro-environmental behavior” and “proenvironmental behavior” were merged into a single canonical form). In the fifth stage, records with missing keyword data were excluded from conceptual analyses, reducing the thematic dataset to 915 publications. The complete keyword standardization dictionary—listing all variant forms, their canonical equivalents, and the merge rationale—is provided as Supplementary Table S1 to enable full reproducibility of the co-occurrence analyses.

#### 2.4.1 Database complementarity analysis

To quantify the degree of overlap and complementarity between WoS and Scopus, we conducted a structured database comparison analysis. Of the 934 unique records, 540 (57.8%) appeared in both databases, 172 (18.4%) were exclusive to WoS, and 222 (23.8%) were exclusive to Scopus. WoS records (*n* = 712 total, including shared records) exhibited a higher mean citation count (40.5; median = 12) and a more recent mean publication year (2019; median = 2021) compared to Scopus-only records (mean citations = 20.3, median = 5; mean year = 2017, median = 2020). These differences are consistent with WoS's more selective indexing policy and its tendency to over-represent high-impact, English-language outlets. The Scopus-only records disproportionately represent recent publications and regional journals—precisely the segments that WoS-only analyses would systematically omit. This confirms that dual-database coverage is methodologically essential for comprehensive bibliometric mapping in interdisciplinary fields (Mongeon and Paul-Hus, 2016).

A methodological consideration concerns the asymmetry between Web of Science and Scopus search architectures. Web of Science Topic (TS) searches include titles, abstracts, author keywords, and Keywords Plus, whereas Scopus TITLE-ABS-KEY searches are restricted to titles, abstracts, and author keywords. Diagnostic inspection identified 39 records (4.2% of the final dataset) that contained Keywords Plus terms without author keywords, indicating that Web of Science indexing modestly expanded retrieval beyond the search-field structure available in Scopus. To minimize database-specific retrieval effects, records from both databases were merged, deduplicated, and subjected to overlap and sensitivity analyses.

### 2.5 Data selection process

The data selection process was documented following PRISMA 2020 reporting principles, adapted for bibliometric methodology. The identification stage yielded 1,474 records. After duplicate removal, 934 records were retained for bibliometric analysis. Ambiguous records and uncertain duplicate cases were resolved through consensus-based review by the research team. Nineteen records were subsequently excluded from conceptual analyses due to missing keyword data, producing a final thematic dataset of 915 publications. This process is summarized in Figure A1.

### 2.6 Analytical framework

All analyses were conducted in R (version 4.3). The core analytical package was bibliometrix (Aria and Cuccurullo, 2017), supplemented by ggplot2 for visualization, dplyr and tidyr for data processing, and igraph for network construction.

#### 2.6.1 Performance analysis

Performance analysis evaluated: (a) annual scientific production and publication growth trajectory; (b) most productive and cited sources; (c) most productive authors; (d) most cited documents; and (e) country-level production and collaboration. The 2026 data point was excluded from trend analysis because it represents an incomplete year (data extracted on 6 April 2026); it is retained in visualizations but interpreted as a partial observation, and trend conclusions are anchored on the 2025 peak.

#### 2.6.2 Network analysis

Two network analyses were performed. A keyword co-occurrence network was constructed in which nodes represent keywords and edges represent co-occurrence frequency. A country collaboration network was constructed in which nodes represent countries and edges represent co-authorship linkages. Node size is proportional to frequency; edge weight is proportional to connection strength.

#### 2.6.3 Thematic mapping

Thematic mapping followed the Callon centrality–density framework (Callon et al., 1991; Cobo et al., 2011; Aria and Cuccurullo, 2017). Centrality (C) indexes a theme's degree of connectedness with other themes in the field; density (D) indexes its internal cohesion. The two dimensions define four quadrants: motor themes (high C, high D), basic/transversal themes (high C, low D), niche themes (low C, high D), and emerging/declining themes (low C, low D).

#### 2.6.4 Thematic evolution analysis

Thematic evolution analysis examined keyword continuity and discontinuity across successive time periods, using inter-period Jaccard similarity to quantify thematic persistence (Cobo et al., 2011). A minimum Jaccard similarity threshold of 0.10 was applied to filter weak inter-period keyword linkages; connections below this threshold were excluded from the evolution flow diagram to prevent visual overplotting and ensure that only substantive thematic continuities were represented. Four periods were defined: 1993–2000, 2001–2010, 2011–2020, and 2021–2026. Period boundaries were determined by two criteria applied conjointly: (1) inflection points in the cumulative annual production curve, where the growth rate shifted qualitatively (identified by visual inspection and corroborated by a first-difference plot of annual counts); and (2) alignment with major disciplinary and policy events—the emergence of sustainability science as a recognized academic discipline around 2000, the consolidation of pro-environmental behavior as a distinct construct around 2010, and the acceleration of SDG-aligned research after 2019. This approach is consistent with best practice in thematic evolution bibliometrics (Cobo et al., 2011; Mukherjee et al., 2022). The empirical plausibility of the selected thematic evolution periods was additionally inspected using a first-difference plot of annual scientific production (Figure S2).

#### 2.6.5 Bibliometric laws

Bradford's Law was applied to assess source concentration by ranking journals by publication output and partitioning them into three equal-output zones. The Bradford multiplier (*n*) was estimated empirically, and zone membership ratios were compared against the classical 1:*n*:*n*^2^ prediction (Bradford, 1934; Biglu et al., 2011). Lotka's Law was applied to assess author productivity distribution using a power-law model: *f* (*x*) = *Cx*^−β^. The exponent β was estimated by least-squares regression; goodness-of-fit was assessed using the Kolmogorov–Smirnov (KS) test. A residual analysis compared observed and theoretically expected author frequency proportions (Lotka, 1926; Price, 1963).

#### 2.6.6 Keyword sensitivity analysis

Thresholds of ≥3, ≥5, and ≥7 co-occurrences were selected to represent low, moderate, and conservative filtering levels, respectively; this range is consistent with thresholds used in comparable bibliometric studies (Mukherjee et al., 2022; Donthu et al., 2021) and was chosen to assess whether thematic conclusions are sensitive to progressively stricter frequency cutoffs.

To assess the robustness of thematic conclusions to the choice of minimum co-occurrence threshold, sensitivity analyses were conducted at three thresholds: ≥3, ≥5, and ≥7 co-occurrences. Keyword network size and core thematic cluster membership were evaluated at each threshold. The same five core themes were retained across all tested thresholds, although their quadrant classification became more threshold-sensitive under stricter frequency filtering (Table S2).

#### 2.6.7 Three-field plot

A three-field (Sankey) plot visualized structural linkages among the three fields of countries, keywords, and journals, illuminating the tripartite knowledge flow architecture of the field.

### 2.7 Ethical considerations

This study used exclusively published bibliographic metadata. No human participants were involved, no personal data were processed, and no experimental interventions were undertaken. Research integrity, transparency, and reproducibility principles were observed throughout.

## 3 Results

### 3.1 Annual scientific production

The environmental awareness–ecological behavior literature spans four decades, from 1981 to 2026. Annual output remained minimal through the 1990s, began a modest increase in the early 2000s, and entered a phase of exponential growth from approximately 2016 onwards (Figure 1). Peak production occurred in 2025, with 132 publications—a figure based on complete annual records, as data extraction was performed on 6 April 2026 and all 2025 publications were therefore fully indexed at the time of retrieval. Publication activity increased substantially over the study period, particularly after 2010, with annual output reaching its peak in 2025 (*n* = 132). The 2026 data point (*n* = 38 at time of extraction) represents a partial year and should not be interpreted as evidence of a declining trend.

**Figure 1:**
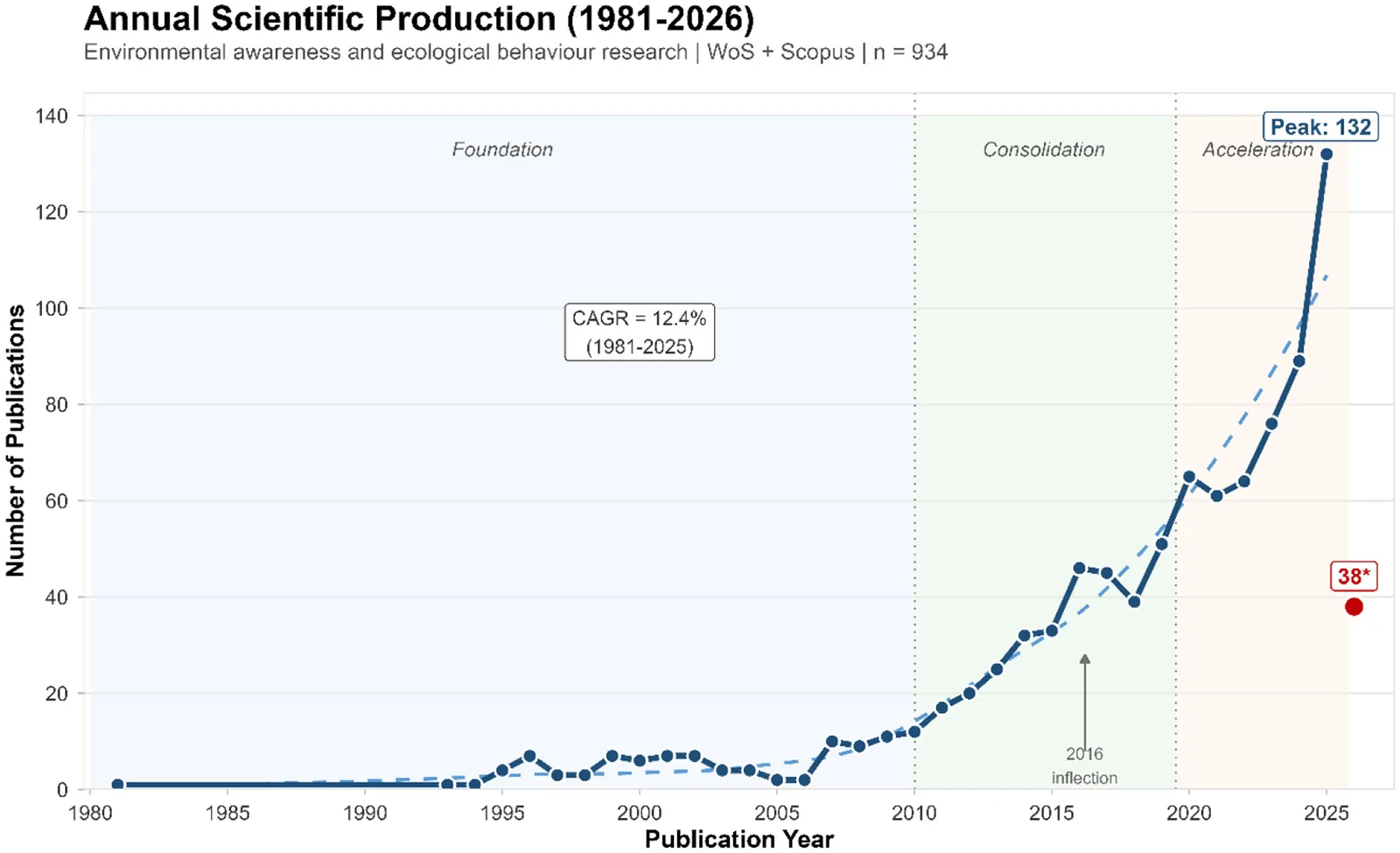
Annual scientific production (1981–2026). The 2026 data point represents an incomplete year. The 2025 count (*n* = 132) is treated as fully indexed at the date of retrieval (6 April 2026), though minor late-indexing effects for publications submitted in the final weeks of 2025 cannot be entirely excluded.

### 3.2 Source analysis and Bradford's law

Publications were distributed across 520 unique source journals. The 15 most productive sources are presented in Table 1 and illustrated in Figure 2. Sustainability led with 37 publications, followed by Journal of Environmental Psychology (*n* = 27) and Journal of Cleaner Production (*n* = 24).

**Table 1:** Top 15 most productive sources.

Rank	Source	Publications
1	Sustainability	37
2	Journal of Environmental Psychology	27
3	Journal of Cleaner Production	24
4	Environmental Education Research	16
5	International Journal of Consumer Studies	15
6	Journal of Environmental Protection and Ecology	12
7	Australian Journal of Environmental Education	11
8	Business Strategy and the Environment	10
9	Current Issues in Tourism	10
10	Environment Development and Sustainability	10
11	Journal of Sustainable Tourism	10
12	Psyecology	9
13	Environmental Conservation	7
14	International Journal of Environment and Sustainable Development	6
15	International Journal of Environmental Research and Public Health	6

Ranked by publication count in the cleaned bibliometric dataset.

**Figure 2:**
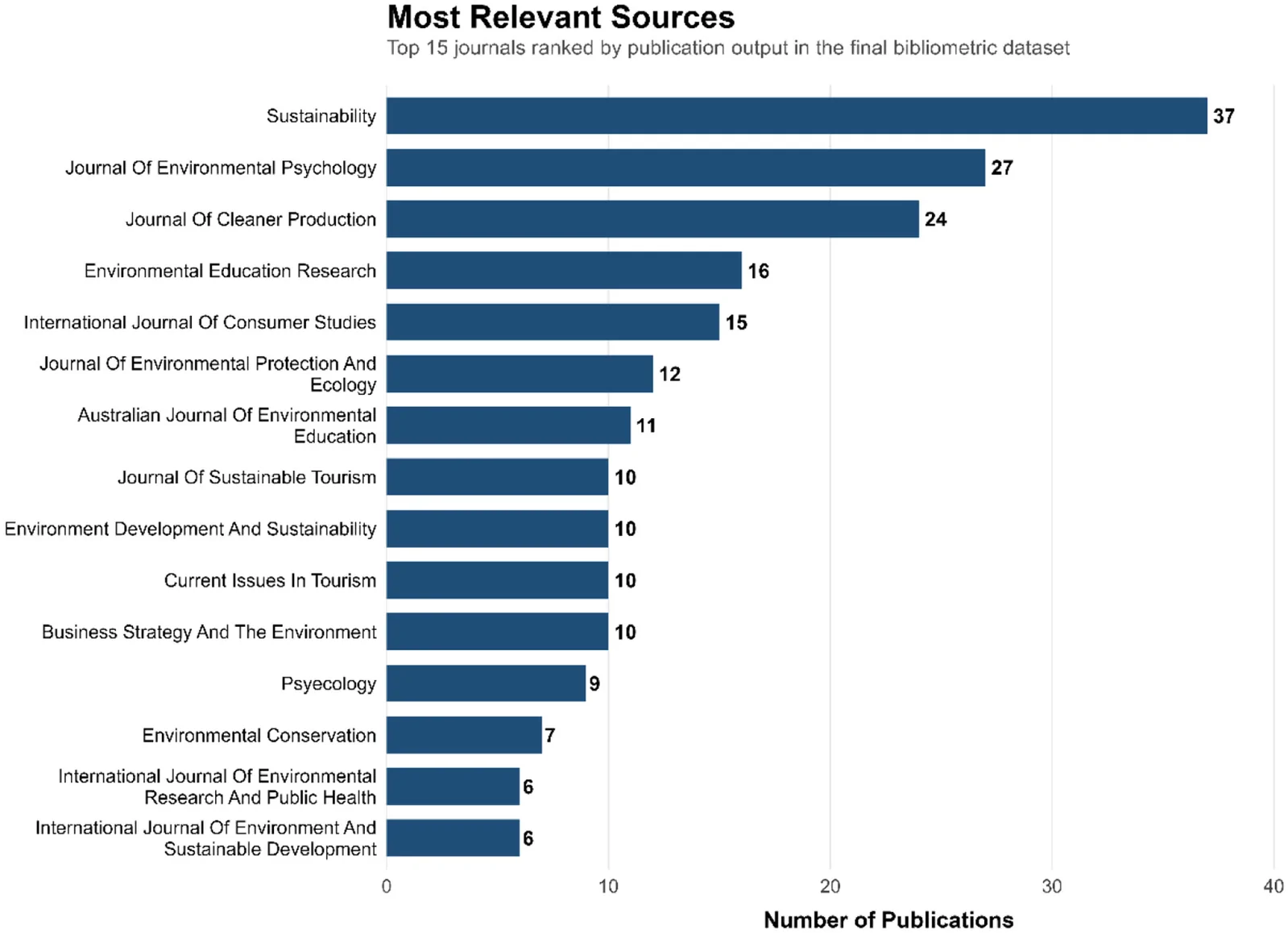
Top 15 most productive sources by publication count. Sustainability leads with 37 publications, reflecting the dominance of open-access multidisciplinary outlets in this field.

Bradford's Law analysis partitioned sources into three equal-output zones (Table A2). Zone 1 comprised 39 journals carrying 310 publications (33.2%); Zone 2 comprised 173 journals with 316 publications (33.8%); Zone 3 comprised 308 journals with 308 publications (33.0%). The minor deviations from strict equal-output partitioning are consistent with the inherent approximation properties of Bradford's asymptotic model. The empirically observed Bradford multiplier ratio was 39:173:308, equivalent to approximately 1:4.4:7.9, deviating from the classical geometrical prediction of 1:*n*:*n*^2^. The Bradford cumulative distribution curve is presented in Figure 3.

**Figure 3:**
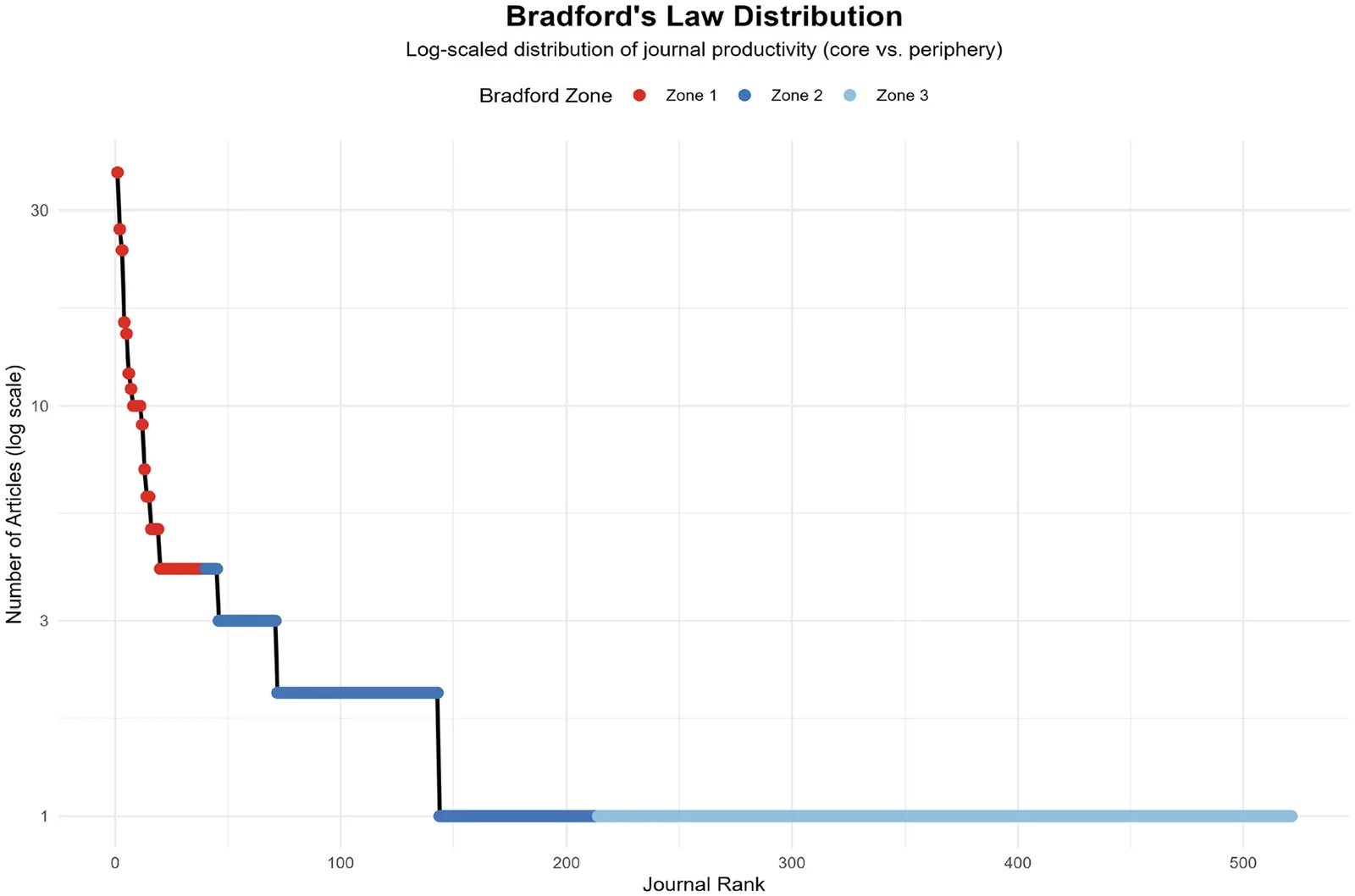
Bradford's Law: log-scale distribution of journal productivity and core-periphery demarcation. The shallower-than-predicted Zone 3 gradient (1:4.4:7.9) is consistent with the interdisciplinary character of the literature.

### 3.3 Author productivity and Lotka's law

The dataset comprised 2,485 unique authors. The 15 most productive authors are presented in Table 2 and the top 20 are visualized in Figure 4, led by Yusliza M. (*n* = 8), followed by Okumah M. (*n* = 7) and Amerigo M., Bogner F., Liobikiene G., and Muhammad Z. (each *n* = 6). Mean authorship per paper was 3.09; 85.4% of papers were multi-authored.

**Table 2:** Top 15 most productive authors by publication count.

Rank	Author	Publications
1	Yusliza M.	8
2	Okumah M.	7
3	Amerigo M.	6
4	Bogner F.	6
5	Liobikiene G.	6
6	Muhammad Z.	6
7	Nguyen N.	5
8	Nguyen T.	5
9	Ogiemwonyi O.	5
10	Ahmad A.	4
11	Bin H. A.	4
12	Chen X.	4
13	Chiarella S.	4
14	Colombo S.	4
15	Corraliza J.	4

Ranked by publication count in the cleaned bibliometric dataset.

**Figure 4:**
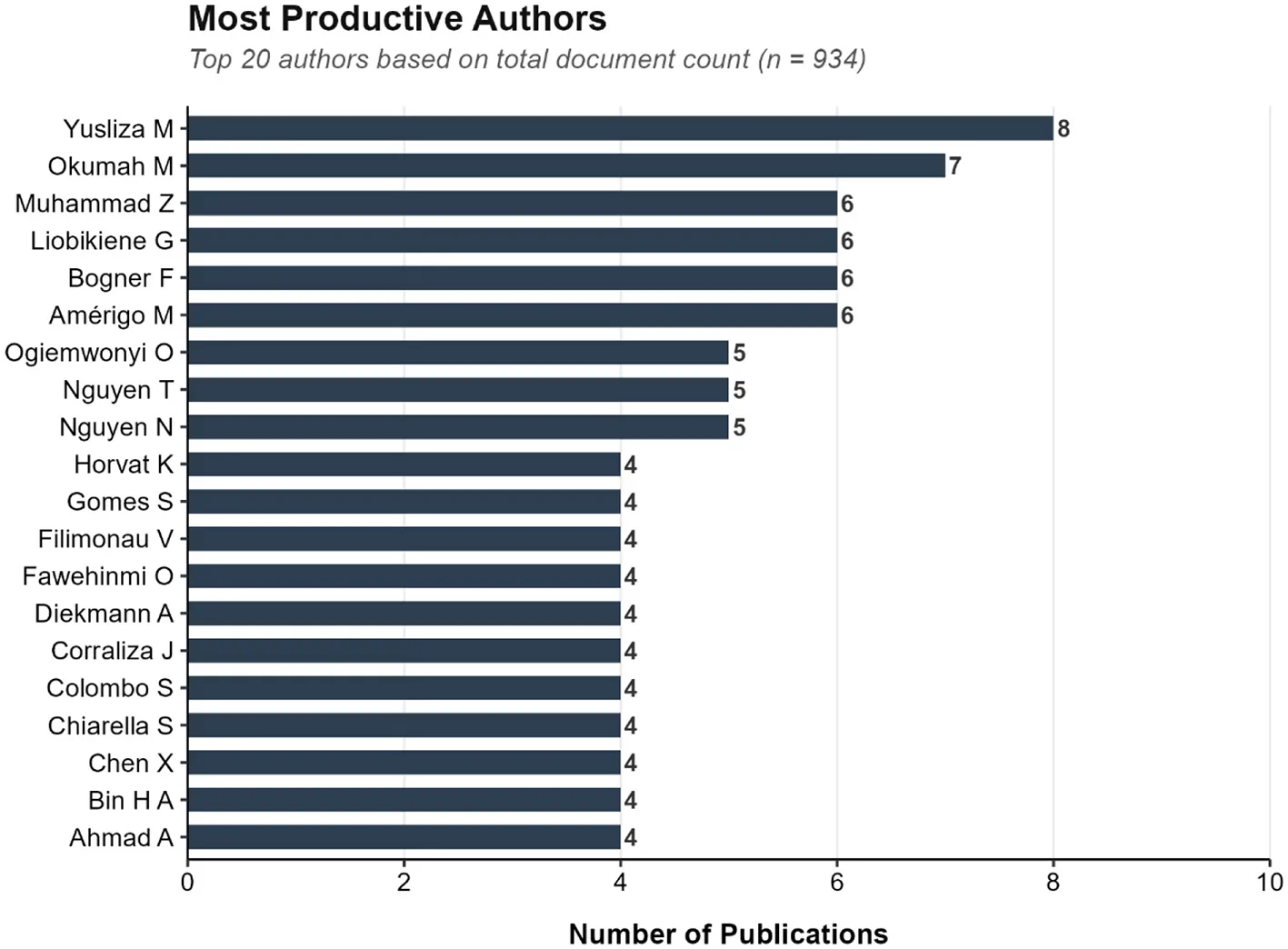
Top 20 most productive authors by publication count.

Lotka's Law analysis revealed that the observed author productivity distribution deviates from the classical inverse-square model (β = 2). The Lotka exponent estimated from the empirical data was β = 3.8345 (C = 1.045; *R*^2^ = 0.9821), indicating a steeper power-law decline than the classical prediction and confirming that the field's authorial structure is more heavily skewed toward single-publication contributors than the inverse-square model anticipates. The two-sample Kolmogorov–Smirnov test comparing the empirical distribution to the theoretical distribution under the classical β = 2 yielded *p* = 0.0879, which does not reach statistical significance at the conventional α = 0.05 threshold. This result indicates insufficient power to formally reject the null hypothesis that the distribution follows the classical β = 2 model; it does not confirm conformity with β = 2. The evidence for departure from the classical model rests instead on the substantially higher empirically estimated exponent (β = 3.8345) and the excellent fit of the estimated model (*R*^2^ = 0.9821), which together indicate that the field's authorial structure is more accurately characterized by a steeper power-law than the inverse-square model predicts. The residual analysis (Table A1) quantifies this imbalance. Expected proportions were derived by evaluating *f* (*x*) = *Cx*^−^β at *x* = 1, 2, …, 8 using the estimated parameters (*C* = 1.045, β = 3.8345), then normalizing the resulting values to sum to unity. Single-publication authors (88.94%) substantially exceeded the theoretical expectation under the fitted model (65.47%), yielding a positive residual of +0.2347. Authors with two or more publications were correspondingly under-represented relative to theoretical predictions, consistent with a field that is broad and participatory but whose stable core of recurrent contributors remains comparatively limited. Figure 5 displays the observed and fitted distributions.

**Figure 5:**
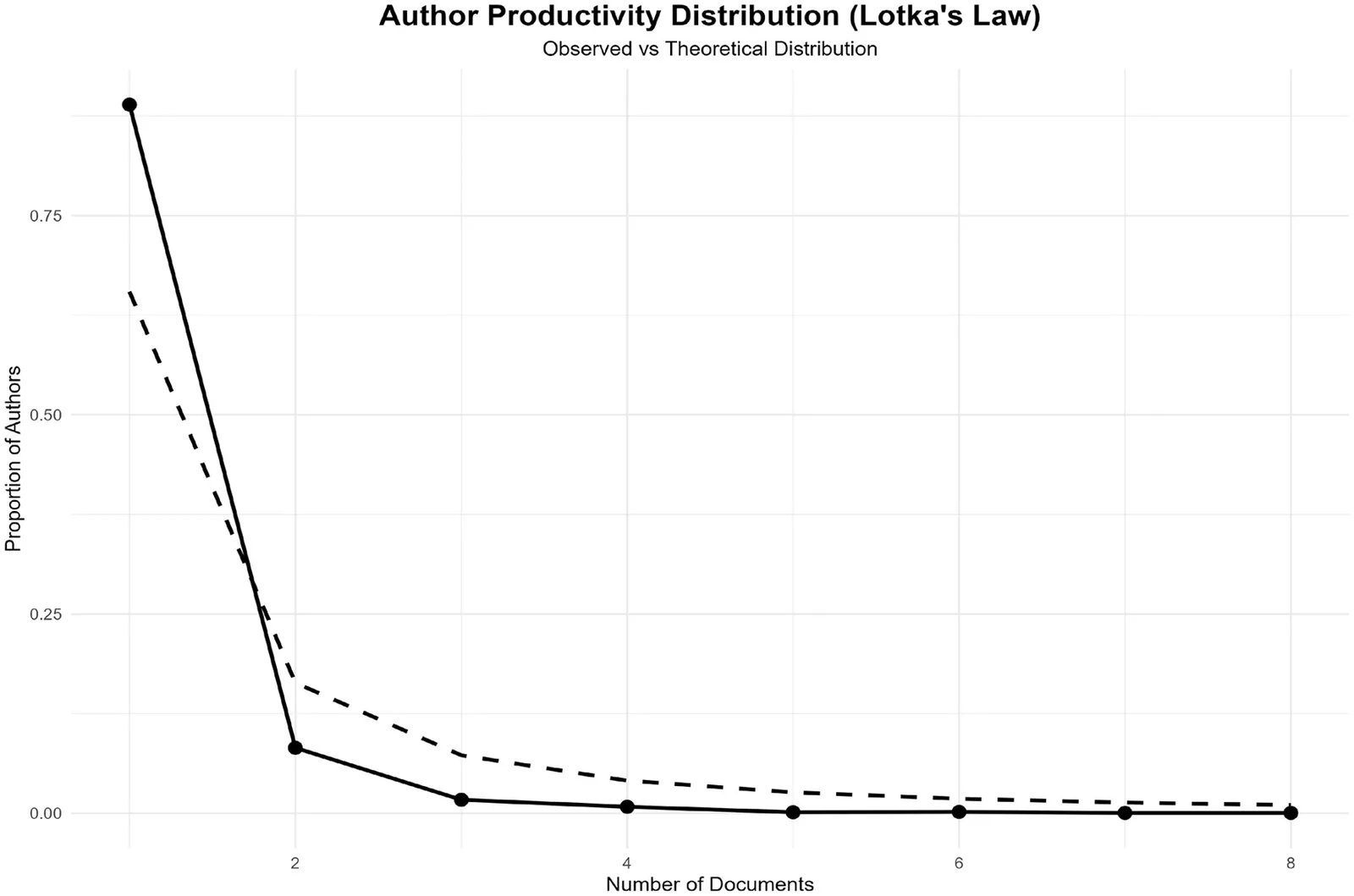
Lotka's Law: observed vs. theoretically expected author productivity distribution (β = 3.8345, *R*^2^ = 0.9821).

### 3.4 Most cited documents

The 15 most cited publications are presented in Table 3 and illustrated in Figure 6. The most cited work was Gifford and Nilsson (2014), with 1,571 citations, followed by Kaiser et al. (1999) with 874 citations, and Lee (2008) with 547 citations. The citation distribution is highly right-skewed, reflecting the cumulative advantage dynamics characteristic of mature scientific literatures (Price, 1965).

**Table 3:** Top 15 most cited documents in the environmental awareness–ecological behavior corpus.

Rank	First author	Year	Title (abbreviated)	Journal	Citations
1	Gifford	2014	Personal and social factors that influence pro-environmental concern and behavior	*Int. J. Psychol*.	1,571
2	Kaiser et al.	1999	Environmental attitude and ecological behavior	*J. Environ. Psychol*.	874
3	Lee	2008	Opportunities for green marketing: young consumers	*Marketing Intel. Plan*.	547
4	Vicente-Molina et al.	2013	Environmental knowledge and other variables affecting pro-env. behavior	*J. Clean. Prod*.	524
5	Diekmann and Preisendörfer	2003	Green and greenback: behavioral effects of env. attitudes	*Ration. Soc*.	518
6	Nisbet et al.	2011	Happiness is in our nature: exploring nature-relatedness	*J. Happiness Stud*.	486
7	Otto and Pensini	2017	Nature-based environmental education of children	*Glob. Environ. Change-H&P*	477
8	Gadenne et al.	2011	Influence of consumers' env. beliefs and attitudes on energy-saving	*Energy Policy*	452
9	Mackay and Schmitt	2019	Do people who feel connected to nature do more to protect it?	*J. Environ. Psychol*.	390
10	Lumber et al.	2017	Beyond knowing nature: contact, emotion, compassion, meaning, and beauty	*PLoS ONE*	388
11	Lu and Wu	2007	Env. principles applicable to green supplier evaluation	*Int. J. Prod. Res*.	328
12	Donald et al.	2014	An extended TPB model of commuters' transport mode use	*J. Environ. Psychol*.	322
13	Grob	1995	A structural model of environmental attitudes and behavior	*J. Environ. Psychol*.	313
14	Miller et al.	2015	Sustainable urban tourism: understanding visitor pro-env. behaviors	*J. Sustain. Tour*.	312
15	Milfont and Duckitt	2004	The structure of environmental attitudes	*J. Environ. Psychol*.	311

Citation counts reflect the merged WoS–Scopus dataset; where both databases reported counts for the same document, the higher value was retained to maximize coverage. Counts have not been normalized by publication age. Titles abbreviated for display; full titles available in the Supplementary data file.

**Figure 6:**
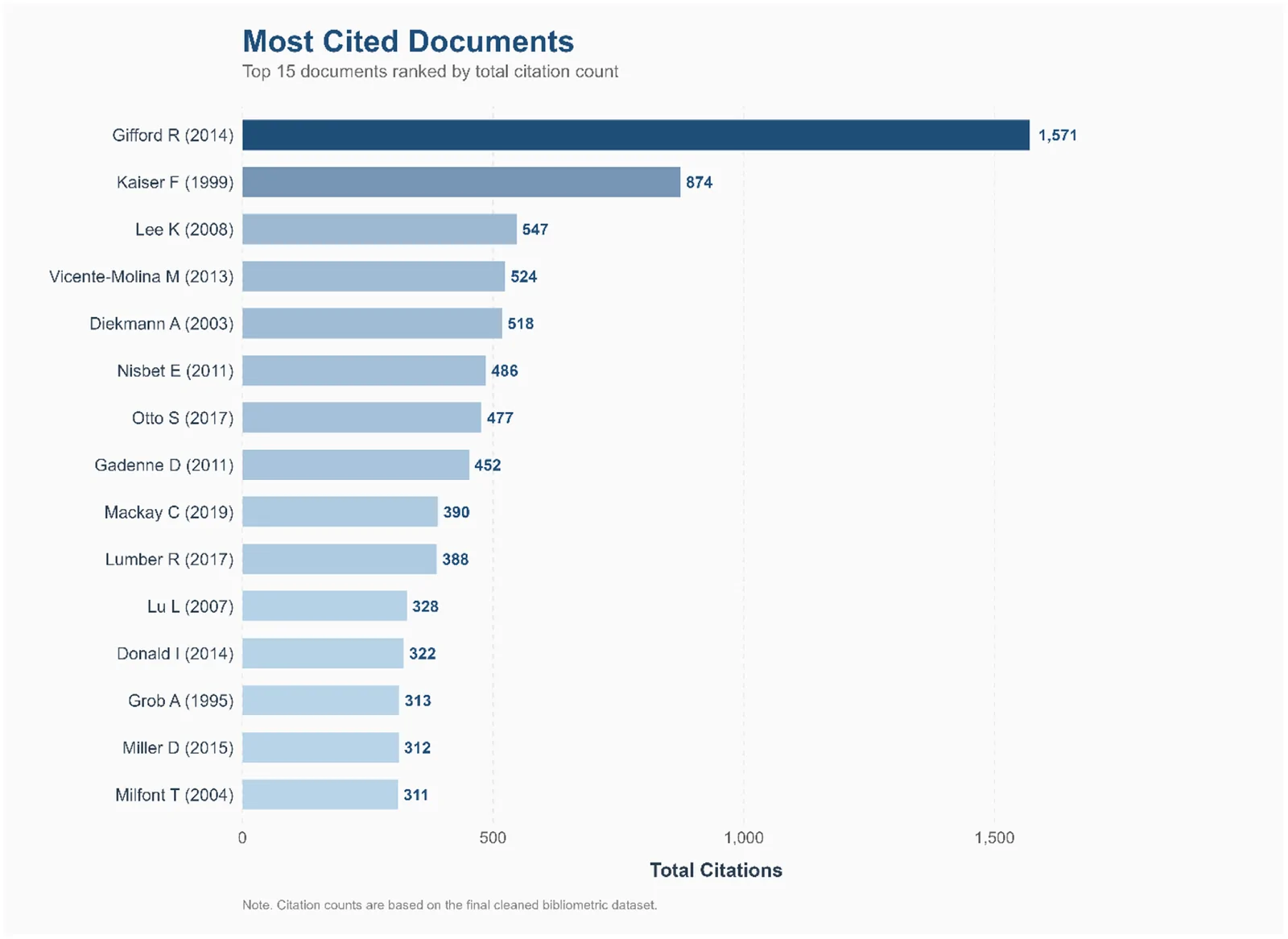
Top 15 most cited documents: citation counts.

### 3.5 Country-level production and collaboration

Country-level analysis identified the United Kingdom as the leading producer (*n* = 126 publications), followed by Malaysia (*n* = 82), Spain (*n* = 80), India (*n* = 69), and Australia (*n* = 68). Figure 7 presents production by country. The country collaboration network (Figure 8) reveals that the United Kingdom occupies the most central collaborative position, with strong ties to the United States, Spain, Canada, China, Australia, and Germany. The network structure is hub-and-spoke rather than fully distributed, indicating a partial concentration of collaborative capital.

**Figure 7:**
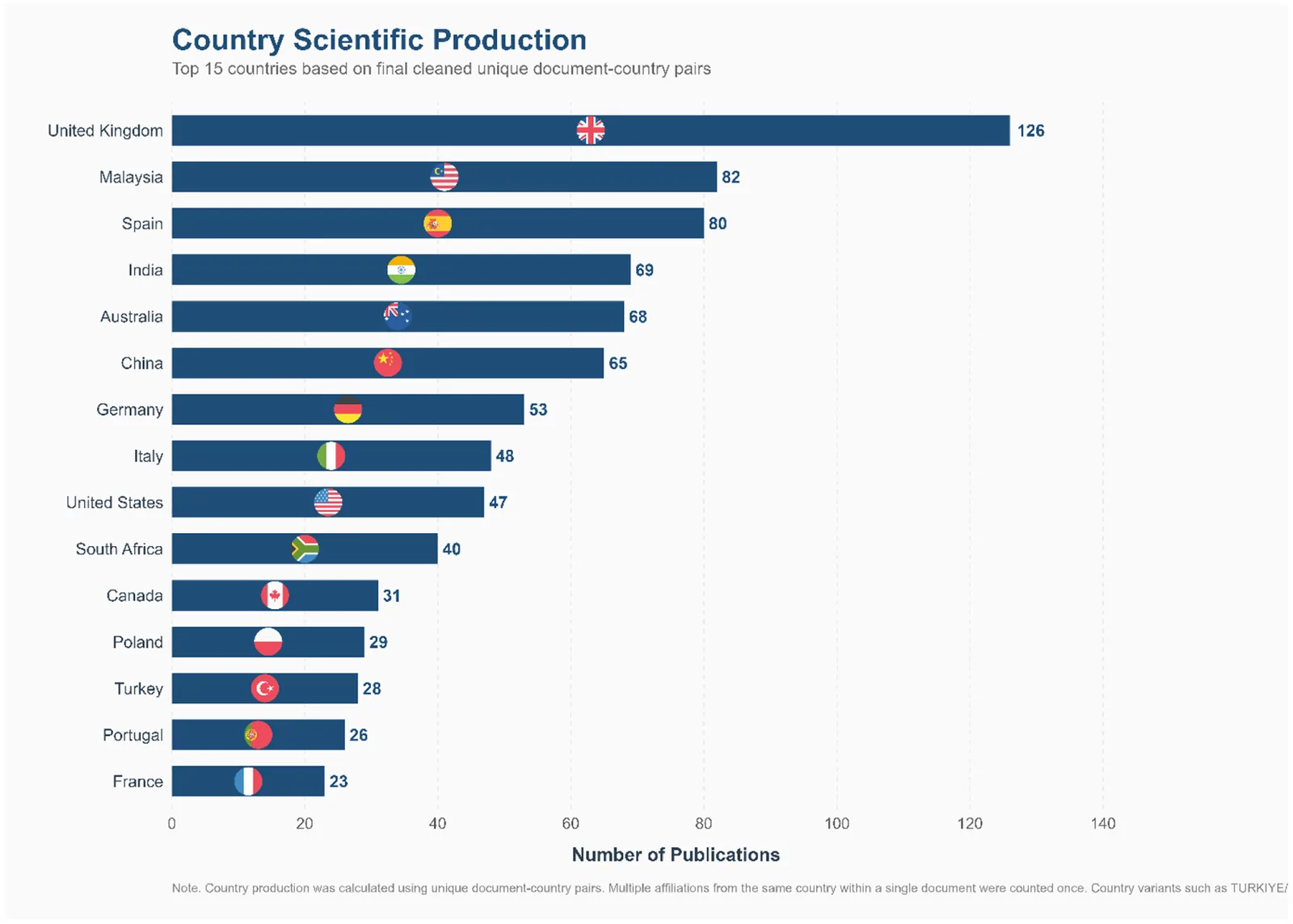
Country-level scientific production: top 15 countries by publication count.

**Figure 8:**
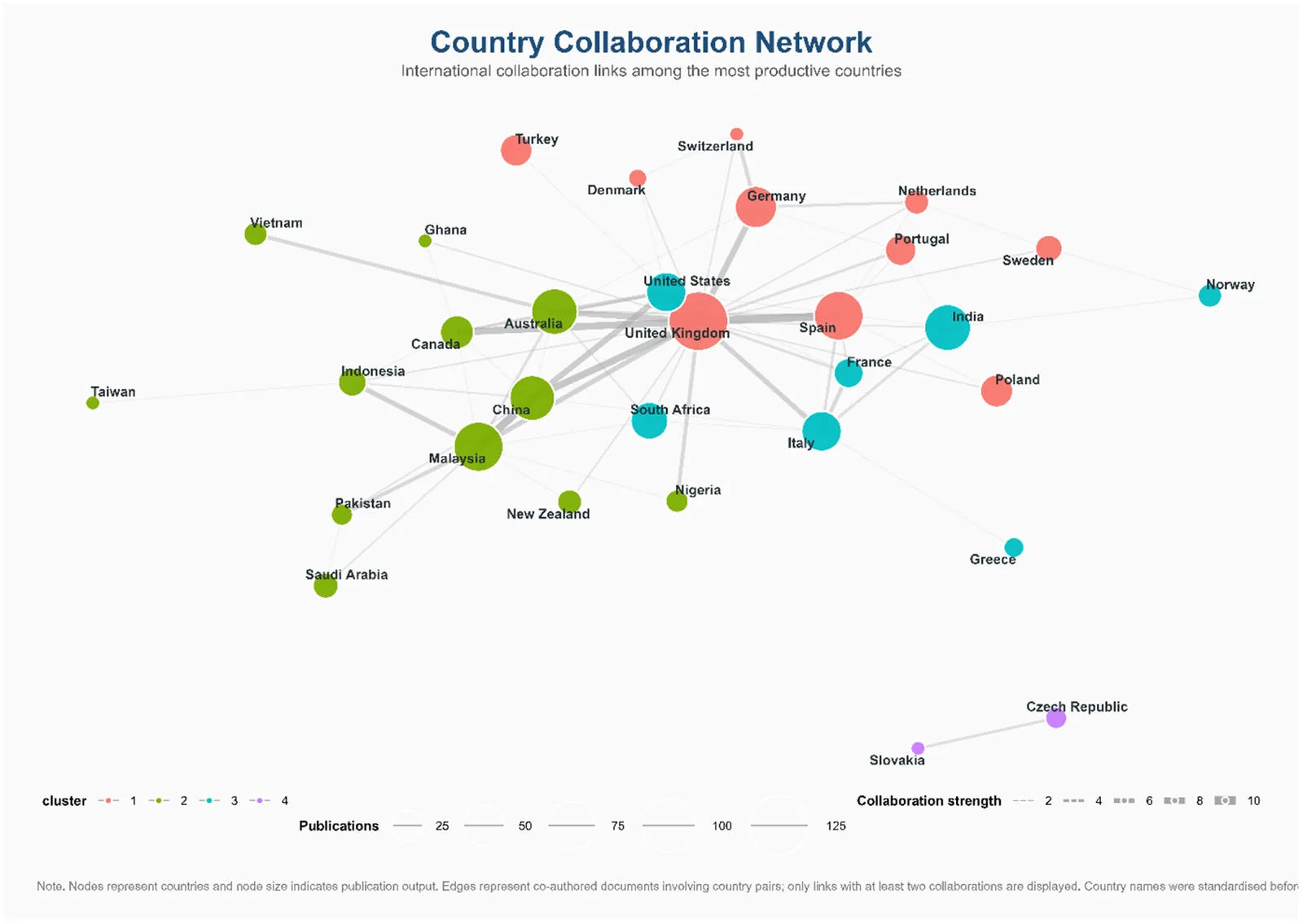
Country collaboration network: co-authorship linkages among productive countries (minimum co-authorship threshold: 2 documents). Node size reflects publication output; edge thickness reflects collaboration strength.

### 3.6 Keyword analysis and robustness

The 15 most frequent keywords are presented in Table 4. “Pro-environmental behavior” (*n* = 287) dominated the keyword space, followed by “attitudes” (*n* = 236), “environmental attitudes” (*n* = 150), “environmental concern” (*n* = 135), and “environmental behavior” (*n* = 134). Figure 9 displays the frequency distribution.

**Table 4:** Top 15 most frequent author keywords by co-occurrence frequency.

Rank	Keyword	Frequency
1	Pro-environmental behavior	287
2	Attitudes	236
3	Environmental attitudes	150
4	Environmental concern	135
5	Environmental behavior	134
6	Values	128
7	Behavior	112
8	Sustainability	108
9	Environmental knowledge	105
10	Planned behavior	95
11	Determinants	94
12	Knowledge	87
13	Environmental awareness	81
14	Environmental education	76
15	Climate change	75

Keywords have been standardized and merged to reduce semantic fragmentation. Some entries (e.g., “Planned behavior,” “Behavior”) retain original American English database spellings; spelling variants were merged as canonical British English forms in all co-occurrence analyses.

**Figure 9:**
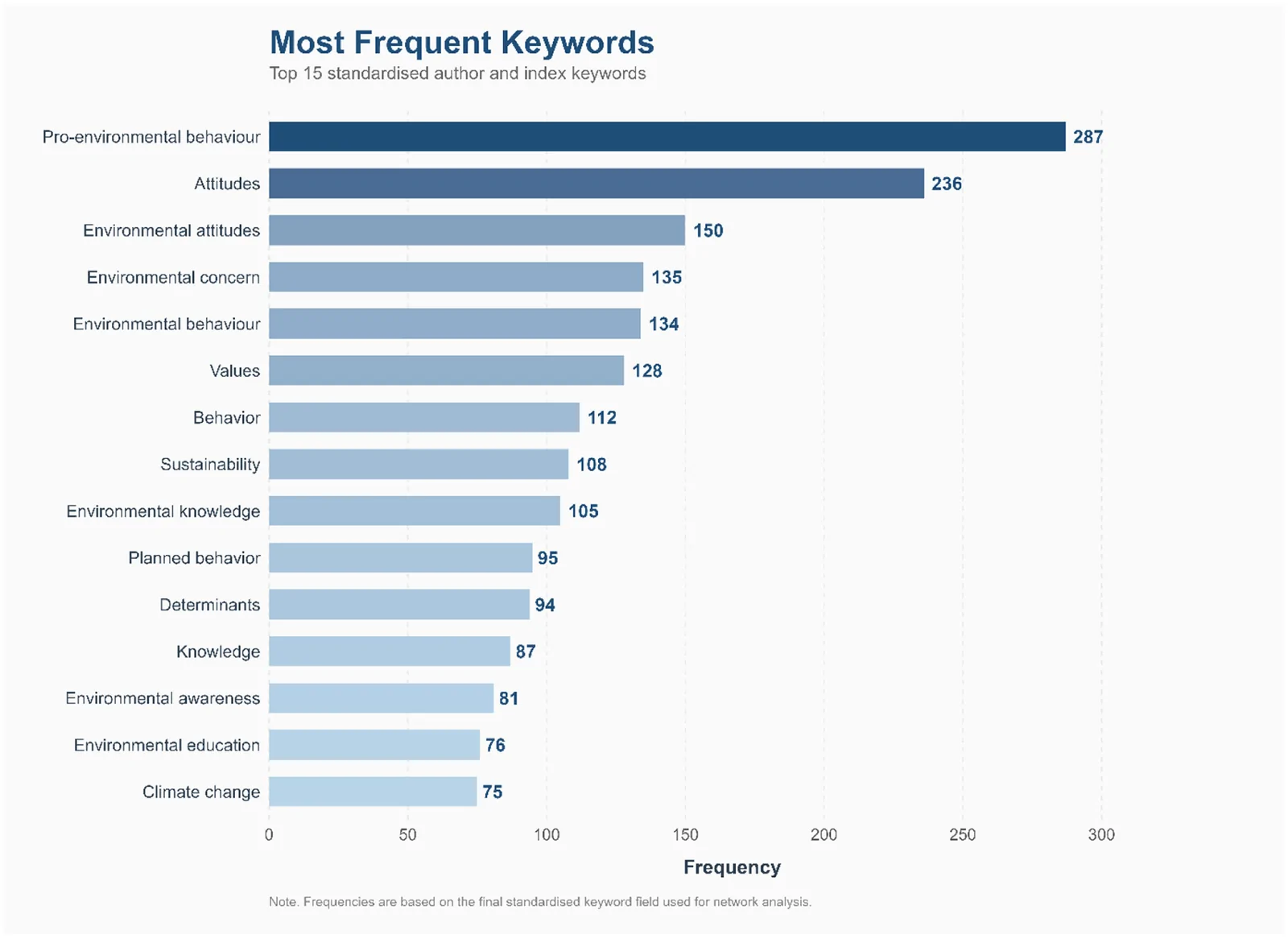
Most frequent keywords: bar chart of top 15 keywords (standardized and merged dataset). Pro-environmental behavior dominates the keyword space (*n* = 287), confirming its role as the integrative construct of the field.

Keyword sensitivity analysis (Table A3; Figure S1) confirmed that the thematic structure is highly robust. At a threshold of ≥3 co-occurrences, 231 keywords were retained in the co-occurrence network. The keyword co-occurrence network (Figure 10) reveals at ≥5, the network comprised 104 keywords; at ≥7, 64 keywords. Across all three conditions, the five core thematic anchors—pro-environmental behavior, attitudes, environmental attitudes, environmental concern, and sustainability—remained consistently central, indicating that the reported thematic conclusions are not artifacts of threshold selection.

**Figure 10:**
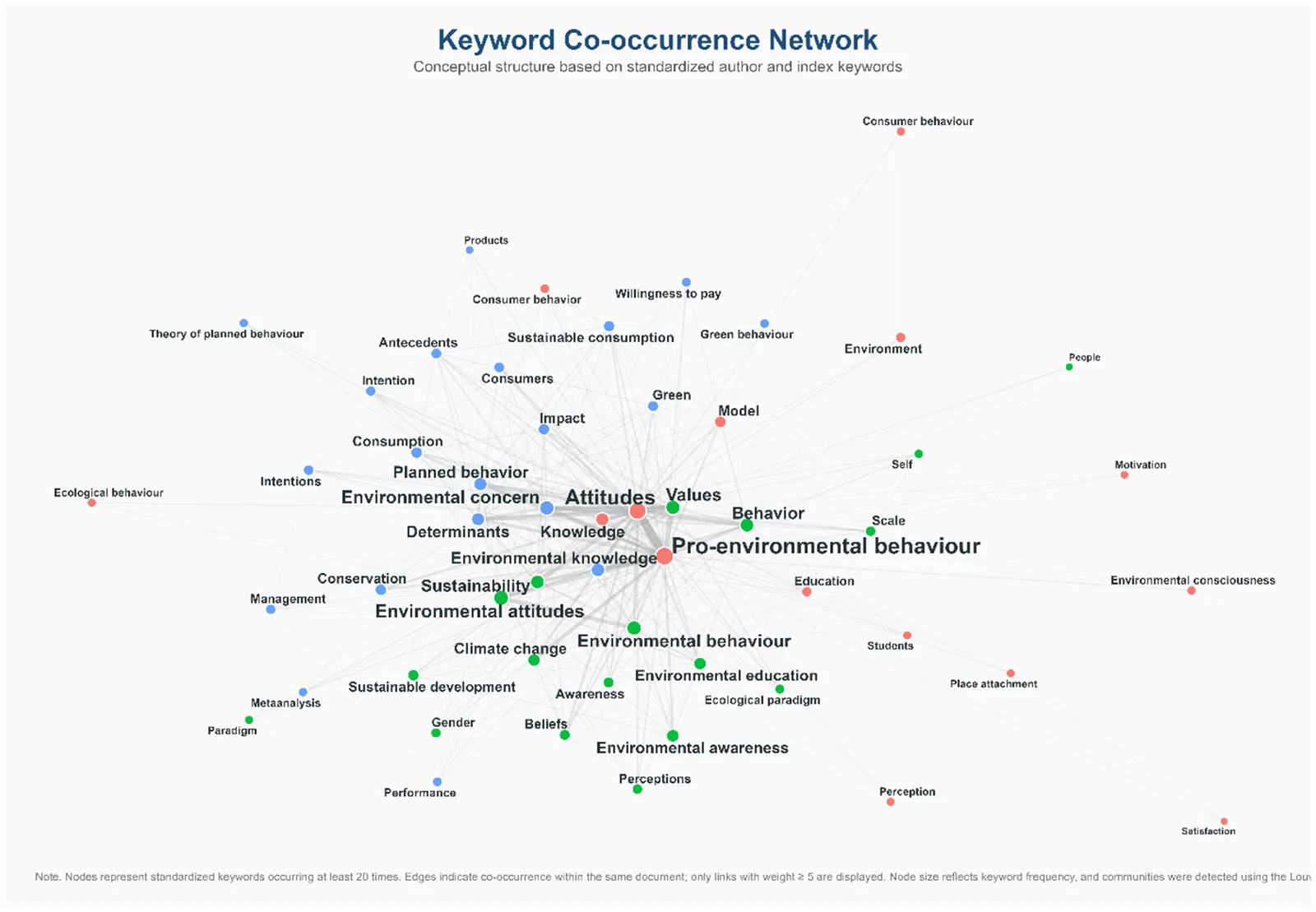
Keyword co-occurrence network: core conceptual structure. Pro-environmental behavior functions as the central hub, maintaining co-occurrence ties to virtually all other high-frequency constructs.

### 3.7 Three-field plot

The three-field (Sankey) plot (Figure 11) reveals the structural interconnections among countries, keywords, and journals. The United Kingdom, China, Spain, and Australia are disproportionately linked to the core keywords (pro-environmental behavior, planned behavior, environmental concern) and to the core source journals (Journal of Cleaner Production, Journal of Environmental Protection and Ecology, Journal of Environmental Psychology). This tri-partite alignment indicates that knowledge production in this field is architecturally organized rather than randomly distributed.

**Figure 11:**
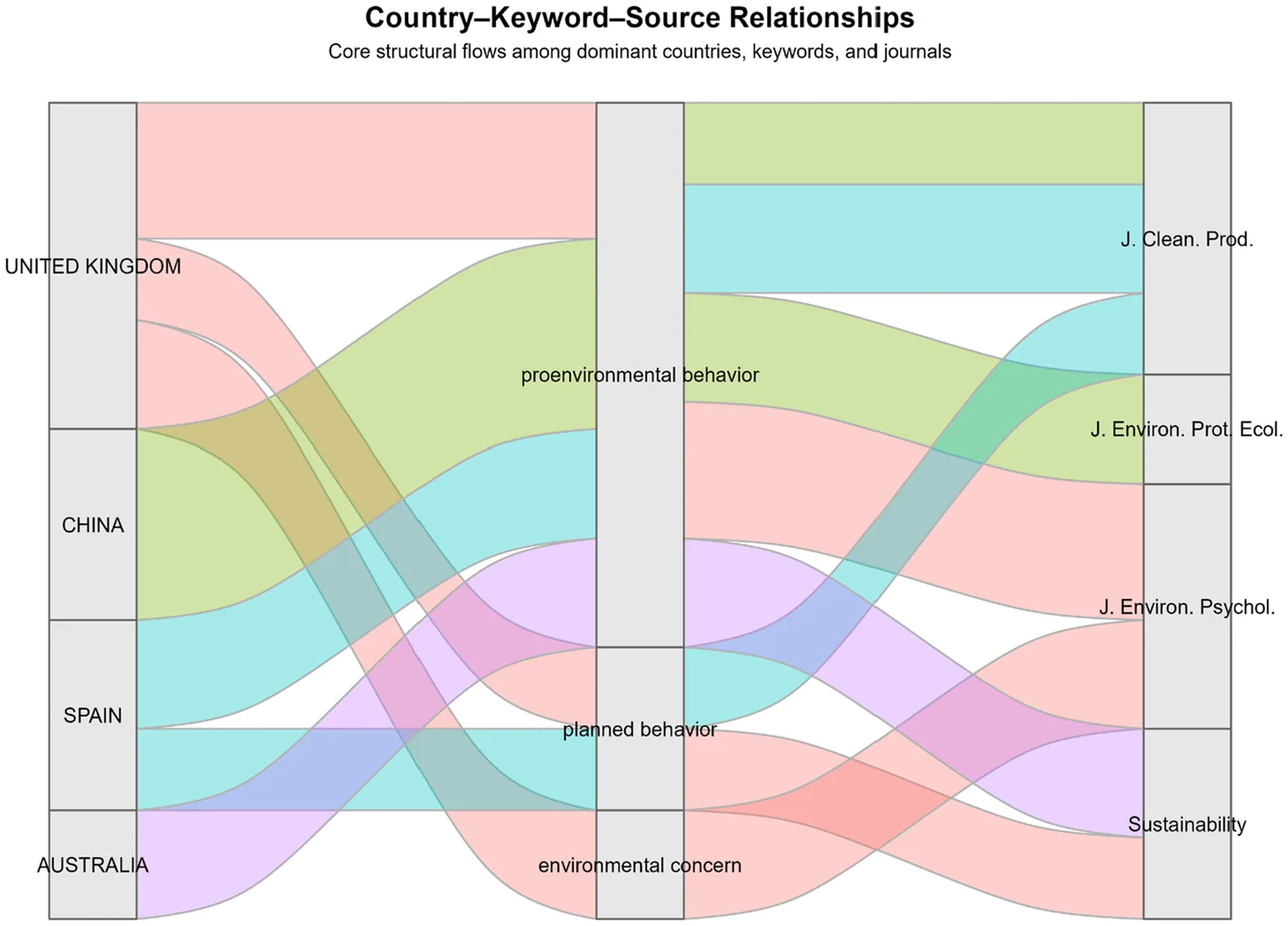
Three-field plot (Sankey diagram): structural linkages among countries, keywords, and journals.

### 3.8 Thematic evolution and mapping

Thematic evolution analysis (Figure 12) examined keyword transitions across four analytical periods (1993–2000, 2001–2010, 2011–2020, 2021–2026). For interpretive purposes, the first two periods (1993–2000 and 2001–2010) are consolidated into a single foundational phase (pre-2010), yielding three qualitatively distinct developmental phases. In the foundation period (1993–2000), publications were sparse and the dominant conceptual anchors were “environmental knowledge,” “environmental attitude,” and “ecological behavior,” reflecting the field's origins in environmental psychology and education. The early growth period (2001–2010) saw these constructs deepen, with “environmental concern” gaining increasing prominence. In the consolidation period (2011–2020), “pro-environmental behavior” became the dominant conceptual node, interconnecting attitude, concern, knowledge, and educational constructs within an increasingly behavior-centered framework. In the acceleration period (2021–2026), new thematic elements emerged, including “sustainable development,” “climate change,” “digital behavior,” and cross-cultural applications, while the core behavioral framework retained its centrality.

**Figure 12:**
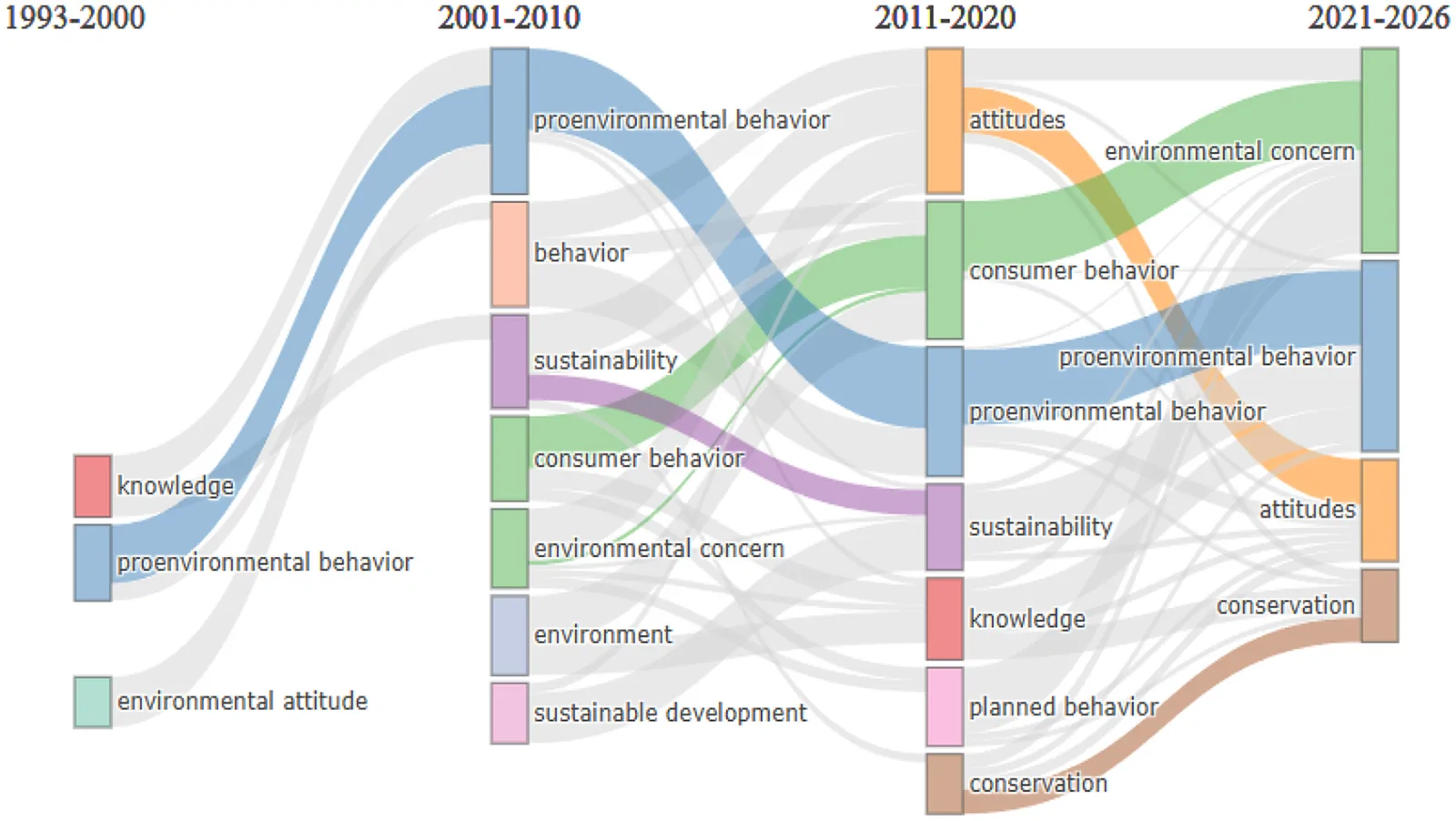
Thematic evolution of the environmental awareness–ecological behavior literature across four periods (1993–2000, 2001–2010, 2011–2020, 2021–2026). The flows indicate the continuity and transition of dominant themes between consecutive periods. The final period (2021–2026) includes 2026, which represents an incomplete publication year.

Thematic mapping (Figure 13) identified four thematic quadrants based on Callon centrality and density scores. “Pro-environmental behavior” was classified in the niche theme quadrant (low centrality, high density), indicating a well-developed internal conceptual structure with limited connectivity to adjacent thematic clusters—a pattern likely reflecting the high degree of terminological standardization around this construct rather than genuine conceptual isolation. “Attitudes” functioned as the primary basic/transversal theme (high centrality, low density), acting as a broad structural bridge across the field's disciplinary communities. “Sustainability” was also positioned in the niche quadrant, reflecting internally cohesive but peripherally connected development. “Planned behavior” was positioned at the boundary of basic and motor themes, consistent with its central theoretical role as a widely deployed scaffold (Ajzen, 1991). “Climate change” occupied the emerging/declining theme position (low centrality, low density), suggesting either nascent thematic integration or gradual peripheralisation relative to the field's dominant behavioral frameworks.

**Figure 13:**
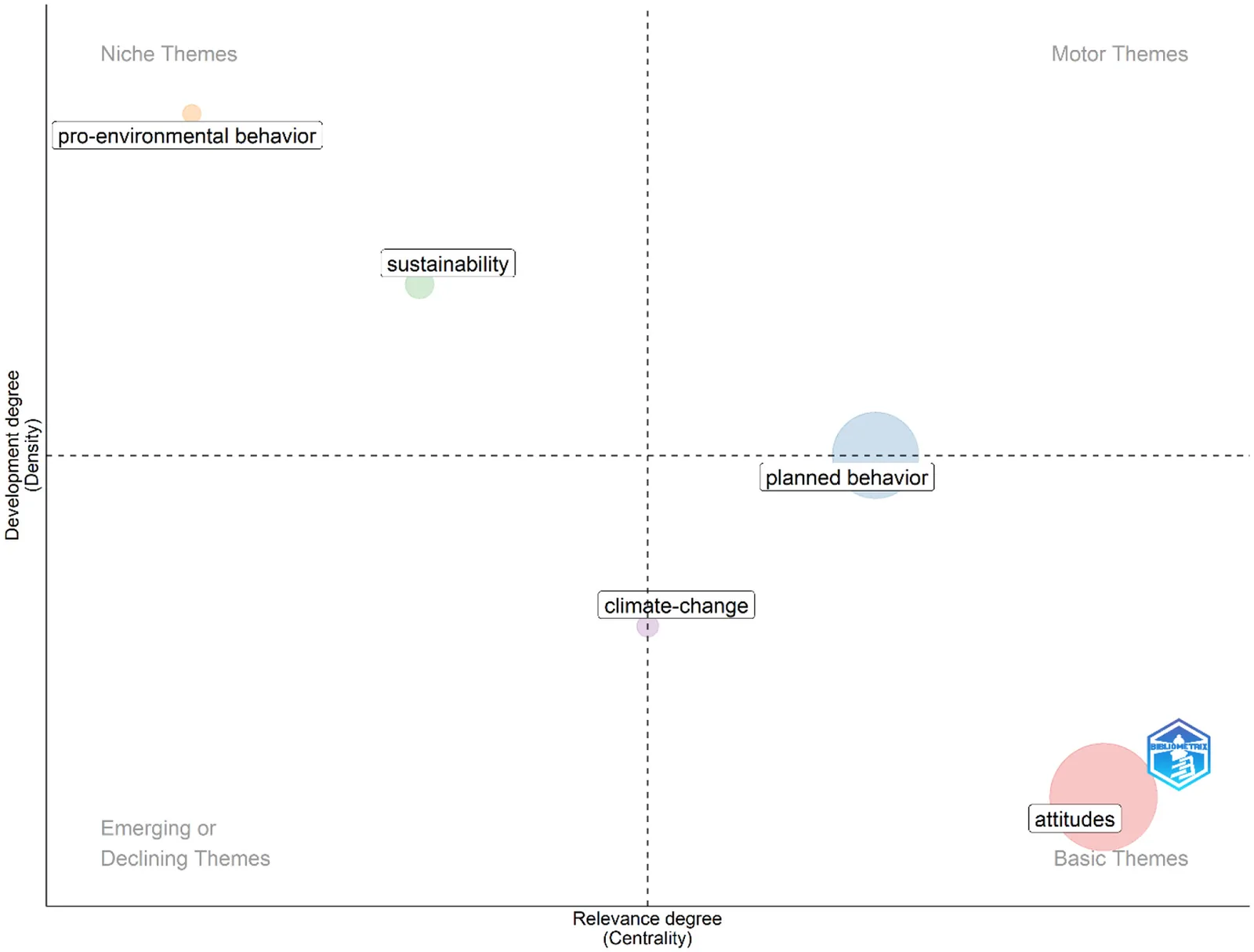
Thematic map: classification of themes by centrality (x-axis) and density (y-axis). Attitudes occupies the basic/transversal quadrant, indicating its role as a structural bridge across disciplinary communities.

## 4 Discussion

This study provides a comprehensive bibliometric portrait of the environmental awareness–ecological behavior research domain. The findings reveal a field that is simultaneously expanding in volume, consolidating in conceptual structure, and diversifying in thematic scope. The following sections interpret these findings through the lenses of bibliometric theory, field development dynamics, and environmental behavior research, integrating the results of database complementarity, Lotka and Bradford law analyses, keyword sensitivity testing, and thematic evolution analysis.

### 4.1 Temporal dynamics and field development

The annual production trajectory documented here—sustained low-level output through the 1990s, moderate growth in the 2000s, and exponential acceleration after 2016—follows the pattern described by Price (1963)'s exponential growth model of scientific literature. The transition from linear to exponential growth is a reliable marker of field institutionalization: it signals that a research domain has achieved sufficient conceptual coherence, methodological standardization, and journal infrastructure to attract investment from researchers outside its founding disciplinary communities (Price, 1965; Cobo et al., 2011). In the present field, this inflection point coincides with the consolidation of sustainability science as a recognized discipline, the adoption of the UN Sustainable Development Goals (SDGs) in 2015, and the publication of landmark IPCC assessment reports that elevated the policy salience of individual and collective behavioral change (Clayton et al., 2015; Swim et al., 2011). The acceleration phase (2021–2026) may reflect, in part, the additional stimulus of post-COVID sustainability transitions; however, this relationship is not directly demonstrated through the bibliometric data and should be interpreted as a plausible contextual explanation rather than an established causal link (Peng and Bai, 2021). These growth dynamics are broadly consistent with the findings of Zhu et al. (2021), who documented steady production growth in WoS-indexed environmental behavior research from 2002 to 2020 using CiteSpace, and with Goyal and Goyal (2024), who observed a notable acceleration in pro-environmental behavior research following the adoption of the UN SDGs. The present study extends these observations by providing a longer temporal window (1981–2026), a dual-database design, and a more granular periodisation that distinguishes three qualitatively distinct developmental phases. The first-difference plot provides additional descriptive support for the increasing growth trajectory of the field, particularly after 2010 and 2020 (Figure S2). However, exponential growth also carries structural risks: rapid publication acceleration in interdisciplinary fields is frequently accompanied by topic saturation, methodological repetition, and publication inflation, wherein the volume of empirical output expands faster than the field's capacity to synthesize and theoretically integrate its findings (Mukherjee et al., 2022). Whether the current growth trajectory represents genuine knowledge accumulation or disciplinary dilution remains an open question that future meta-analytic work should address.

### 4.2 Database complementarity

The WoS–Scopus complementarity analysis produced findings with direct methodological implications for bibliometric research in this domain. The 222 Scopus-exclusive records (23.8% of the cleaned dataset) represent a non-trivial disciplinary segment that would have been systematically omitted in a single-database study. The lower mean citation count of Scopus-exclusive records (20.3 vs. 40.5 for WoS records) reflects, in part, their more recent mean publication year (2017 vs. 2019): citation accumulation is a time-dependent process, and newer publications structurally receive fewer citations irrespective of quality (Hirsch, 2005; Price, 1965). However, the difference also reflects WoS's selectivity bias toward established, high-impact journals—a selection mechanism that tends to systematically underrepresent regional journals, non-English-language publications, and emerging subdisciplinary outlets (Mongeon and Paul-Hus, 2016). In the environmental awareness–ecological behavior field, this distinction is consequential: substantial contributions originate from education research, tourism studies, and consumer behavior journals that are more comprehensively indexed in Scopus than in WoS. The database complementarity analysis thus confirms a standard recommendation of contemporary bibliometric methodology—dual-database indexing is not a precaution but a necessity for comprehensive mapping in interdisciplinary fields.

### 4.3 Source concentration and Bradford's law

Bradford's Law analysis revealed a clear core–periphery structure, with 39 journals carrying 33.2% of all publications. The empirically observed Bradford multiplier ratio was 39:173:308 (approximately 1:4.4:7.9). Under the classical Bradford geometrical model (1:*n*:*n*^2^) with the empirically estimated multiplier *n* = 4.44 (Zone 2/Zone 1 = 173/39), the classical model would predict Zone 3 to contain approximately 39 × 4.44^2^ ≈ 768 journals, yielding a predicted ratio of approximately 1:4.4:19.7. The observed Zone 3 count (308 journals) is therefore substantially smaller than the classical prediction, indicating a shallower-than-predicted literature dispersion (Bradford, 1934; Biglu et al., 2011). Such deviations are consistent with findings in other interdisciplinary fields and have been attributed to the structural complexity of multi-paradigmatic domains: when a literature draws from multiple parent disciplines (in this case, environmental psychology, education, management, and sustainability science), the transition between core and periphery journals is smoother because peripheral journals are not merely occasional publishers but dedicated outlets of adjacent disciplines (Egghe, 1990).

The specific composition of Zone 1 illuminates the field's disciplinary geography. The co-presence of Sustainability, Journal of Environmental Psychology, and Journal of Cleaner Production as the three most productive outlets may suggest that the field simultaneously inhabits three discursive communities: sustainability science (problem-oriented), environmental psychology (mechanism-oriented), and cleaner production/business (application-oriented). This tripartite structure is consistent with the three-field plot findings and corroborates the thematic map's identification of “pro-environmental behavior” as a transversal theme that bridges disciplinary boundaries. The relative dominance of Sustainability, an open-access multidisciplinary journal, is consistent with the possibility that the field's growth is partly associated with increased accessibility of publication venues, which may contribute to the broadening of its authorial base. At the same time, the dominance of a broad open-access outlet such as Sustainability in Zone 1 raises a legitimate concern: the breadth of its scope may attract methodologically and conceptually heterogeneous contributions that reduce the coherence of the disciplinary core, and citation concentration in a single multidisciplinary journal may artificially inflate apparent field integration while masking underlying fragmentation.

### 4.4 Author productivity dynamics and Lotka's law

The Lotka's Law residual analysis produced one of the most theoretically informative findings of this study. The observed proportion of single-publication authors (88.94%) exceeded the theoretical expectation under the estimated exponent (β = 3.8345) by a residual of +0.2347—corresponding to approximately 1.36 times the expected value. In the literature on scientometric indicators, a large positive residual for single-publication authors is a diagnostic marker of early-stage or boundary-crossing field development (Price, 1963; Lotka, 1926). It indicates that the field attracts a disproportionately large number of transient contributors—researchers from adjacent disciplines or applied practice communities who engage with the environmental awareness–ecological behavior literature for a single publication cycle before returning to their home disciplines. This structural pattern is consistent with what Price (1963) described as the “invisible college” dynamic in frontier sciences—permeable knowledge boundaries that simultaneously attract diverse perspectives and impede the formation of a stable, programme-driven authorial core. Such fragmentation carries direct epistemic costs: fields dominated by transient contributors tend to accumulate isolated empirical findings that are difficult to synthesize, replicate, or build upon cumulatively (Lotka, 1926; Price, 1963; Mukherjee et al., 2022).

### 4.5 Intellectual structure and citation patterns

The citation distribution of the most influential documents exhibits the classic right-skewed pattern of cumulative advantage (Price, 1965): the top five cited works—Gifford and Nilsson (2014), Kaiser et al. (1999), Lee (2008), Vicente-Molina et al. (2013), and Diekmann and Preisendörfer (2003)—collectively account for a disproportionate share of total citations. Of these five, Lee (2008); Vicente-Molina et al. (2013), and Diekmann and Preisendörfer (2003) are identified here as the most-cited documents within the bibliometric corpus itself (Table A1) rather than as direct citations of this manuscript, and are therefore not separately listed in the References section. This distribution is consistent with the theoretical architecture of the field: these works represent foundational syntheses and empirical benchmarks in the attitude–behavior relationship (Kaiser et al., 1999), the multidimensional determinants of pro-environmental concern (Gifford and Nilsson, 2014), and the knowledge–behavior gap (Kollmuss and Agyeman, 2002). Their persistent citation centrality suggests that the field's theoretical core has been relatively stable for over two decades—a characteristic of mature paradigmatic structures in the Kuhnian sense (Kuhn, 1962). At the same time, the emergence of more recent works (Geiger et al., 2019; Hernández Alemán et al., 2024; Wan and Du, 2022) in the mid-citation band indicates that the field is generating second-generation empirical contributions that refine rather than replace the foundational frameworks.

### 4.6 Thematic structure and keyword robustness

The keyword sensitivity analysis provides a methodological quality guarantee that is rarely reported in bibliometric studies but is epistemologically essential. The finding that the thematic structure—anchored on pro-environmental behavior, attitudes, environmental attitudes, environmental concern, and sustainability—remained fully intact across co-occurrence thresholds of ≥3, ≥5, and ≥7 indicates that the reported thematic conclusions are not threshold artifacts. At ≥7, the network was reduced to 64 keywords (from 231 at ≥3), yet the five core thematic nodes remained the highest-centrality items. This stability implies that the conceptual architecture of the field is genuinely robust and not sensitive to analytical parameterisation—a criterion that Mukherjee et al. (2022) identify as fundamental to bibliometric validity.

The co-occurrence network itself reveals a non-random conceptual architecture. “Pro-environmental behavior” functions as the integrative hub, maintaining co-occurrence ties to virtually all other high-frequency keywords. This structural position is consistent with the construct's theoretical role as the dependent variable or outcome variable of the research programme—all antecedent and contextual constructs are connected to the network through their relationship to behavioral outcomes (Bamberg and Möser, 2007; Steg and Vlek, 2009). The strong co-occurrence of “planned behavior” (*n* = 95) within this network confirms that Ajzen (1991)'s Theory of Planned Behavior has been extensively applied as a theoretical framework for linking attitudes, norms, and intentions to behavior, making it one of the most deployed theoretical scaffolds in the field. The continued presence of “environmental education” as a high-frequency keyword (*n* = 76) indicates that educational interventions—as opposed to purely psychological or marketing-based approaches—represent a distinct and persistent strand of the literature (Zsóka et al., 2013).

### 4.7 Thematic evolution: from awareness to behavior

The thematic evolution analysis provides the most theoretically consequential finding of this study: a systematic, directional shift from awareness-centered to behavior-centered to mechanism- and intervention-centered inquiry across the three developmental phases. This trajectory mirrors the broader arc of environmental social science, which has progressively moved from documenting the “attitude–behavior gap” (Kollmuss and Agyeman, 2002) to explaining why the gap persists (Gifford, 2011; Lorenzoni et al., 2007) and, most recently, to designing conditions that close it (Abrahamse and Steg, 2013; Dietz et al., 2009).

The thematic map (Figure 13) places “attitudes” as the primary basic/transversal theme—a structurally central but internally diffuse construct that bridges environmental psychology, consumer behavior, and sustainability communities. This bridging function is consistent with decades of research establishing attitudinal constructs as foundational antecedents of pro-environmental action (Kaiser et al., 1999; Bamberg and Möser, 2007). “Planned behavior” is positioned at the boundary of basic and motor themes, reflecting the field-level dominance of Ajzen (1991)'s Theory of Planned Behavior (TPB)—which links attitude, subjective norm, and perceived behavioral control to behavioral intentions—as a core theoretical scaffold. These findings are broadly consistent with Value–Belief–Norm theory (Stern, 2000), which connects values and norms through affective concern constructs. Taken together, attitudinal and normative predictors dominate the co-occurrence network, helping explain why the literature has progressively converged on social norm activation—rather than information provision alone—as the primary lever for pro-environmental action (Nordlund and Garvill, 2002; de Groot and Steg, 2008; Bamberg and Möser, 2007).

### 4.8 Geographical structure and knowledge inequality

The country-level analysis documents a significant geographical concentration of knowledge production. The United Kingdom's position as the leading producer—with central collaborative ties to the United States, Spain, Canada, China, Australia, and Germany—reflects a hub-and-spoke collaboration network in which a small number of institutionally well-resourced countries mediate the majority of international knowledge exchange. This structure is consistent with core–periphery dynamics in global science (Price, 1963) and raises concerns about systematic underrepresentation of Global South perspectives in the field's conceptual agenda (Mongeon and Paul-Hus, 2016; Henrich et al., 2010). Environmental behavior research conducted primarily in WEIRD (Western, Educated, Industrialized, Rich, Democratic) contexts risks generating theoretical frameworks and behavioral mechanisms of limited cross-cultural transferability (Henrich et al., 2010). Henrich et al. (2010) demonstrated that psychological findings derived predominantly from WEIRD samples—precisely the profile of much existing pro-environmental behavior research—are systematically unrepresentative of global human variability in values, norms, and behavioral dispositions. In the context of environmental behavior, this implies that the causal pathways linking environmental concern to PEB, which have been largely established in Northern European, North American, and East Asian (principally Malaysian and Chinese) samples, may not generalize to Sub-Saharan African, Latin American, or South Asian contexts where the institutional enablers of individual pro-environmental action differ fundamentally (van der Linden, 2015; Ntanos et al., 2019). The near-absence of African and Latin American countries from the collaboration network is not merely a bibliometric curiosity; it is a theoretical risk that undermines the universalist aspirations of the field's dominant frameworks. The prominence of Malaysia in the collaboration network is noteworthy and may reflect the growth of sustainability-oriented research agendas in Southeast Asian academic institutions—a trend that has not yet been systematically documented in the bibliometric literature and constitutes a productive direction for future investigation.

### 4.9 Synthesis: a structurally consolidating field

Taken together, the bibliometric evidence presented in this study characterizes the environmental awareness–ecological behavior field as exhibiting four defining structural properties. First, the field is growing exponentially, with production dynamics consistent with Price (1963)'s model of scientific frontier expansion. Second, publication and citation patterns are consistent with structural consolidation around a limited number of core journals, a stable theoretical vocabulary and a persistent intellectual core. Third, the field is authorially diffuse, with a large transient contributor periphery that broadens disciplinary reach at the cost of cumulative authorial investment. Fourth, thematic evolution from awareness- and knowledge-centered inquiry toward mechanism-, intervention-, and application-oriented research may suggest theoretical development, though confirming theoretical maturation would require content-level analysis beyond bibliometric indicators. These patterns are reminiscent of what Price (1963) described as the transition from exploratory frontier growth toward more institutionalized knowledge production—though whether this trajectory will consolidate into a normative science stage remains an empirical question that future content analyses and citation network studies would need to address (Mukherjee et al., 2022; Zupic and Cater, 2015).

## 5 Conclusion

This study has provided, to our knowledge, the first dual-database, law-validated, sensitivity-tested bibliometric mapping of the environmental awareness–ecological behavior literature. Using a dataset of 934 unique publications drawn from WoS and Scopus, and employing performance analysis, network analysis, bibliometric law validation, thematic mapping, and keyword robustness testing, it has generated a multi-dimensional structural portrait of one of the most active interdisciplinary research programmes in contemporary sustainability science.

These findings have direct implications for both researchers and practitioners. For researchers, the thematic map and evolution analysis identify several high-priority gaps: the nature connectedness–behavior nexus, digital pro-environmental behavior, cross-cultural generalization of Western behavioral models, and the integration of structural enablers with individual-level interventions. For practitioners and policymakers, the persistent centrality of environmental concern as a motor theme suggests that affective engagement—not merely information provision—should anchor public communication and educational intervention strategies. For bibliometric methodologists, the database complementarity and keyword sensitivity analyses presented here demonstrate that these robustness checks are not optional quality enhancements but structural components of credible bibliometric scholarship. In terms of research design, future studies should prioritize longitudinal and mixed-methods designs that integrate bibliometric mapping with systematic content analysis, thereby moving beyond structural characterization toward theory-building. Regarding interdisciplinary collaboration, the three-field plot identifies the United Kingdom, China, Spain, and Australia as the most structurally central nodes in the global collaboration network; targeted partnerships between these hubs and underrepresented Global South contributors—particularly from Latin America and Sub-Saharan Africa—represent the most promising avenue for broadening both the empirical base and the cultural generalisability of environmental behavior research.

## 6 Limitations and future research directions

This study has several limitations that bound the generalisability of its conclusions. First, the analysis is restricted to articles and review papers indexed in WoS and Scopus; book chapters, conference proceedings, gray literature, and non-indexed regional journals—which may carry important contributions, particularly from Global South contexts—are excluded. Second, bibliometric analysis characterizes structural and relational properties of the literature but cannot evaluate theoretical depth, methodological quality, or conceptual validity of individual works. Third, keyword standardization, while performed systematically, inevitably involves judgements that could affect co-occurrence network topology; the sensitivity analysis demonstrates that the core structure is robust, but peripheral cluster configurations may vary across standardization choices. Fourth, thematic evolution periods were defined *a priori* on the basis of production trajectory analysis; alternative periodisation schemes might yield different inter-period transition patterns. Fifth, the 2026 data represent a partial year and are treated conservatively throughout. Sixth, while 2025 annual output (*n* = 132) is treated as fully indexed at the date of retrieval (6 April 2026), minor late-indexing delays for publications submitted or accepted in the final weeks of 2025 cannot be entirely excluded; the reported peak count may therefore marginally underestimate the true annual total. Several additional limitations merit acknowledgment. The analysis was restricted to English-language records; publications in other languages, which may carry substantive regional contributions, are systematically underrepresented in both WoS and Scopus and are therefore absent from this analysis. Author name disambiguation was performed using the built-in author-cleaning function of the bibliometrix package, which clusters variant spellings based on surname similarity and co-authorship overlap; residual errors cannot be entirely ruled out and may marginally affect Lotka productivity estimates. Country attribution followed a full counting rule: every country affiliated with any author on a multi-authored paper was counted once per paper, consistent with established practice in collaboration network analysis; this inflates collaboration counts relative to fractional counting but preserves the full scope of international linkages. Finally, citation counts in Table 3 reflect the merged WoS-Scopus dataset; where both databases reported counts for the same document, the higher value was retained and counts have not been normalized by publication age.

Several directions for future research are indicated by these findings. Quantitative meta-analysis and systematic review should target the specific mechanisms linking environmental concern to PEB—the motor theme identified here—to resolve outstanding questions about mediation and moderation pathways (Hernández Alemán et al., 2024). Cross-cultural comparative studies using equivalent measures are needed to establish the boundary conditions of the behavioral frameworks developed predominantly in Western, educated samples (van der Linden, 2015; Nordlund and Garvill, 2002). Longitudinal bibliometric studies tracking authorial investment, citation velocity, and thematic migration would complement the cross-sectional portrait provided here. Finally, dedicated attention to digital pro-environmental behavior—an emerging theme visible in the acceleration phase—is warranted, as digital contexts introduce new structural constraints and affordances that existing behavioral frameworks do not fully accommodate (Peng and Bai, 2021).

## Data Availability

The original contributions presented in the study are included in the article/Supplementary material, further inquiries can be directed to the corresponding author.

## Supplementary material

Table A1 Lotka's Law Residual Analysis: Observed Versus Theoretically Expected Author Productivity Distribution.

Table A2 Bradford's Law Zone Analysis: Source Distribution Across Three Equal-Output Zones.

Table A3 Keyword Co-occurrence Sensitivity Analysis: Network Metrics Across Three Co-occurrence Thresholds (≥3, ≥5, ≥7).

Table A4 WoS–Scopus Database Complementarity Analysis: Record Overlap and Exclusive Coverage by Database.

Table S1 Keyword Standardisation Dictionary: Original Terms, Standardised Forms, and Merge Decisions.

Table S2 Thematic Map Sensitivity Analysis: Quadrant Assignments Across Co-occurrence Thresholds.

Table S3 Search Strings Used for Record Retrieval in Web of Science Core Collection and Scopus.
