# Supplementary material for: From environmental awareness to ecological behavior: a bibliometric mapping of the literature
Source: Front Res Metr Anal. 2026 Sep 18;11:1906919. doi: 10.3389/frma.2026.1906919 (PMC13630643; doi:10.3389/frma.2026.1906919)
Supplement: Supplementary file 1 [file Supplementary_file_1.docx]

**Appendix - Supplementary Material**

**Table A1. Lotka's Law Residual Analysis**

| **Documents Written** | **Number of Authors** | **Observed (%)** | **Expected (%)** | **Residual** |
| --- | --- | --- | --- | --- |
| 1 | 2211 | 88.94 | 65.47 | +0.2347 |
| 2 | 204 | 8.21 | 16.37 | -0.0816 |
| 3 | 42 | 1.69 | 7.27 | -0.0558 |
| 4 | 20 | 0.80 | 4.09 | -0.0329 |
| 5 | 3 | 0.12 | 2.62 | -0.0250 |
| 6 | 4 | 0.16 | 1.82 | -0.0166 |
| 7 | 1 | 0.04 | 1.34 | -0.0130 |
| 8 | 1 | 0.04 | 1.02 | -0.0098 |

*Note. Estimated Lotka exponent β = 3.8345, R² = 0.9821, p = 0.0879. The p-value refers to the two-sample Kolmogorov–Smirnov test comparing the empirical author productivity distribution with the theoretical Lotka distribution under β = 2 (the classical inverse-square model). Residual = Observed proportion − Expected (β = 3.8345) proportion. The positive residual for single-publication authors indicates a higher-than-expected proportion of occasional contributors.*

**Table A2. Bradford Zone Analysis**

| **Bradford Zone** | **Journals (n)** | **Publications (n)** | **Share (%)** |
| --- | --- | --- | --- |
| Zone 1 (Core) | 39 | 310 | 33.2 |
| Zone 2 | 173 | 316 | 33.8 |
| Zone 3 (Periphery) | 308 | 308 | 33.0 |
| Total | 520 | 934 | 100.0 |

*Note. Bradford zone multiplier ratio: 39:173:308 ≈ 1:4.4:7.9. Classical prediction: 1:n:n².*

**Table A3. Keyword Sensitivity Analysis**

| **Minimum Frequency Threshold** | **Keywords Retained (n)** | **Core Structure Preserved** |
| --- | --- | --- |
| ≥3 | 231 | Yes |
| ≥5 | 104 | Yes |
| ≥7 | 64 | Yes |

*Note. Core themes: pro-environmental behaviour, attitudes, environmental attitudes, environmental concern, sustainability. All core themes remained the highest-centrality nodes at every threshold.*

**Table A4. WoS–Scopus Database Complementarity Analysis**

| **Database** | **Records (n)** | **Mean Year** | **Median Year** | **Mean Citations** | **Median Citations** |
| --- | --- | --- | --- | --- | --- |
| WoS | 712 | 2019 | 2021 | 40.5 | 12 |
| Scopus-only | 222 | 2017 | 2020 | 20.3 | 5 |

*Note. "Scopus-only" refers to the 222 unique Scopus records with no WoS counterpart (23.8% of the cleaned dataset). Citation counts are based on the cleaned bibliometric dataset.*

**Table S1. Keyword Standardization Dictionary**

| **Variant form(s)** | **Canonical form** | **Standardisation rationale** |
| --- | --- | --- |
| pro environmental behavior; pro environmental behaviour; proenvironmental behavior; proenvironmental behaviour; pro-environmental behavior; pro-environmental behaviour | pro-environmental behaviour | Hyphenation, compound-word and spelling standardisation |
| environmental behavior; environmental behaviour | environmental behaviour | American/British spelling harmonisation |
| attitude; attitudes | attitudes | Singular/plural consolidation |
| environmental attitude; environmental attitudes | environmental attitudes | Singular/plural consolidation |
| climate change; climate-change | climate change | Hyphenation standardisation |
| planned behavior; planned behaviour | planned behavior | American/British spelling harmonisation; canonical database form retained |
| sustainable consumption; green consumption | sustainable consumption | Conceptual consolidation of closely related consumption terms |
| environmental knowledge; environment knowledge | environmental knowledge | Lexical standardisation |
| environmental awareness; environment awareness | environmental awareness | Lexical standardisation |
| sustainable development; sustainability development | sustainable development | Lexical standardisation |

*Note. Keyword standardisation was performed before keyword frequency, co-occurrence network, thematic map, and thematic evolution analyses.*

A1. PRISMA 2020 flow diagram: data selection process (Appendix).

*Figure S1. Keyword co-occurrence sensitivity analysis: network structures at ≥3, ≥5, and ≥7 thresholds.*

*Figure S2. First-Difference Plot of Annual Scientific Production. The figure presents year-to-year changes in publication output between 1993 and 2025. The incomplete 2026 publication year was excluded to avoid an artificial negative first difference. Dashed vertical lines indicate the thematic evolution period boundaries used in the study.*

**Table S2. Thematic Map Sensitivity Analysis Across Minimum Keyword Frequency Thresholds**

| **Theme** | **minfreq=3** | **minfreq=5** | **minfreq=7** | **Stability Assessment** |
| --- | --- | --- | --- | --- |
| Attitudes | Motor theme | Motor theme | Niche theme | Stable at lower thresholds; sensitive at minfreq=7 |
| Planned behavior | Motor theme | Motor theme | Niche theme | Stable at lower thresholds; sensitive at minfreq=7 |
| Sustainability | Motor theme | Motor theme | Niche theme | Stable at lower thresholds; sensitive at minfreq=7 |
| Climate change | Motor theme | Motor theme | Niche theme | Stable at lower thresholds; sensitive at minfreq=7 |
| Pro-environmental behavior | Motor theme | Motor theme | Niche theme | Stable at lower thresholds; sensitive at minfreq=7 |

*Note. Theme composition remained stable across thresholds, but quadrant classification was sensitive to the stricter minfreq=7 threshold.*

**Table S3: Complete Search Strings**

**Scopus**:

TITLE-ABS-KEY ( ( "environmental awareness" OR "ecological awareness" OR "environmental consciousness" OR "environmental concern" OR "environmental knowledge" OR "environmental attitude*" ) AND ( "ecological behavio?r" OR "pro-environmental behavio?r" OR "environmental behavio?r" OR "sustainable behavio?r" OR "green behavio?r" ) ) AND ( LIMIT-TO ( DOCTYPE , "ar" ) OR LIMIT-TO ( DOCTYPE , "re" ) )

**Web of Science Core Collection:**

TS = ( ( "environmental awareness" OR "ecological awareness" OR "environmental consciousness" OR "environmental concern" OR "environmental knowledge" OR "environmental attitude*" ) AND ( "ecological behavio?r" OR "pro-environmental behavio?r" OR "environmental behavio?r" OR "sustainable behavio?r" OR "green behavio?r" ) )

Note: Both searches were conducted on 6 April 2026. Document types were restricted to articles (ar) and reviews (re). The WoS search applies document type filtering through the database interface rather than within the query string.
